# Ecological drivers of fine-scale distribution of arbuscular mycorrhizal fungi in a semiarid Mediterranean scrubland

**DOI:** 10.1093/aob/mcad050

**Published:** 2023-03-28

**Authors:** Jesús López-Angulo, Silvia Matesanz, Angela Illuminati, David S Pescador, Ana M Sánchez, Beatriz Pías, Julia Chacón-Labella, Marcelino de la Cruz, Adrián Escudero

**Affiliations:** Área de Biodiversidad y Conservación, Universidad Rey Juan Carlos, 28933, Móstoles, Madrid, Spain; Department of Environmental Systems Science, Swiss Federal Institute of Technology Zurich (ETH), 8092 Zurich, Switzerland; Área de Biodiversidad y Conservación, Universidad Rey Juan Carlos, 28933, Móstoles, Madrid, Spain; Área de Biodiversidad y Conservación, Universidad Rey Juan Carlos, 28933, Móstoles, Madrid, Spain; Área de Biodiversidad y Conservación, Universidad Rey Juan Carlos, 28933, Móstoles, Madrid, Spain; Departamento de Farmacología, Farmacognosia y Botánica, Facultad de Farmacia, Universidad Complutense de Madrid, 28040, Madrid, Spain; Área de Biodiversidad y Conservación, Universidad Rey Juan Carlos, 28933, Móstoles, Madrid, Spain; Departamento de Biodiversidad, Ecología y Evolución, Facultad de Ciencias Biológicas, Universidad Complutense de Madrid, 28040, Madrid, Spain; Departamento de Biología, Universidad Autónoma de Madrid, 28049, Madrid, Spain; Área de Biodiversidad y Conservación, Universidad Rey Juan Carlos, 28933, Móstoles, Madrid, Spain; Área de Biodiversidad y Conservación, Universidad Rey Juan Carlos, 28933, Móstoles, Madrid, Spain

**Keywords:** Above-ground, arbuscular mycorrhizal fungal diversity, AM fungi, below-ground, DNA metabarcoding, phylogenetic relatedness, plant communities, plant–soil interactions, species composition

## Abstract

**Background and Aims:**

Arbuscular mycorrhizal (AM) fungi enhance the uptake of water and minerals by the plant hosts, alleviating plant stress. Therefore, AM fungal–plant interactions are particularly important in drylands and other stressful ecosystems. We aimed to determine the combined and independent effects of above- and below-ground plant community attributes (i.e. diversity and composition), soil heterogeneity and spatial covariates on the spatial structure of the AM fungal communities in a semiarid Mediterranean scrubland. Furthermore, we evaluated how the phylogenetic relatedness of both plants and AM fungi shapes these symbiotic relationships.

**Methods:**

We characterized the composition and diversity of AM fungal and plant communities in a dry Mediterranean scrubland taxonomically and phylogenetically, using DNA metabarcoding and a spatially explicit sampling design at the plant neighbourhood scale.

**Key Results:**

The above- and below-ground plant community attributes, soil physicochemical properties and spatial variables explained unique fractions of AM fungal diversity and composition. Mainly, variations in plant composition affected the AM fungal composition and diversity. Our results also showed that particular AM fungal taxa tended to be associated with closely related plant species, suggesting the existence of a phylogenetic signal. Although soil texture, fertility and pH affected AM fungal community assembly, spatial factors had a greater influence on AM fungal community composition and diversity than soil physicochemical properties.

**Conclusions:**

Our results highlight that the more easily accessible above-ground vegetation is a reliable indicator of the linkages between plant roots and AM fungi. We also emphasize the importance of soil physicochemical properties in addition to below-ground plant information, while accounting for the phylogenetic relationships of both plants and fungi, because these factors improve our ability to predict the relationships between AM fungal and plant communities.

## INTRODUCTION

Arbuscular mycorrhizal (AM) fungi are members of the subphylum Glomeromycotina (Spatafora *et al.*, 2016), which form mutualistic associations with >70 % of plant species (Brundrett and Tedersoo, 2018). AM fungi enhance the uptake of water and mineral nutrients by the hosts (Lambers *et al.*, 2006; Smith and Read, 2010). In return, plant hosts provide carbohydrates to their symbiotic partners (Kiers *et al.*, 2011). Thus, AM interactions play key roles in ecosystem functioning and the provision of services, such as plant growth and carbon sequestration (Van Der Heijden *et al.*, 1998; Zhu and Miller, 2003; Wilson *et al.*, 2009).

Although it is generally assumed that AM fungi–plant associations are non-specific (Brundrett, 2002; Helgason *et al.*, 2007; Öpik *et al.*, 2009), some studies have reported the existence of host plant preferences (Vandenkoornhuyse *et al.*, 2002; Torrecillas *et al.*, 2012). Such preferences would lead to non-random relationships between the diversity and composition of AM fungal communities and the distribution of plant roots in the soil (García de León *et al.*, 2016; Xu *et al.*, 2016; Neuenkamp *et al.*, 2018; Šmilauer *et al.*, 2020). Root sampling at the individual plant level has evidenced the complexity and variability of AM fungi–plant associations (Davison *et al.*, 2015; Šmilauer *et al.*, 2020), revealing a highly heterogeneous distribution of AM fungal communities. The heterogeneous spatial structure of AM fungal communities might be even more obvious in patchy environments (Davison *et al.*, 2012), such as drylands, where a discontinuous distribution of the vegetation is a common feature (Aguiar and Sala, 1999; Maestre and Cortina, 2005). A better understanding of how plant community attributes (including diversity and composition) might affect the spatial structure of the AM fungal communities is particularly important in dry ecosystems because water accessibility and nutrient absorption by plants are often limited, and the AM fungal–plant associations might play a key role alleviating plant stress (Sánchez-Blanco *et al.*, 2004; Barea *et al.*, 2005; Khalvati *et al.*, 2005; Allen, 2007).

Given that vegetation is more easily accessible above-ground than below-ground, most previous studies assessing the AM fungal–plant relationships at the community level have focused on the distribution of vegetation above-ground, although direct interactions take place below-ground. However, a decoupling between above- and below-ground plant community attributes has been observed in different environments (e.g. Jones *et al.*, 2011; Träger *et al.*, 2019; Illuminati *et al.*, 2021), related to different plant community dynamics acting in the two compartments, such as the ability of many perennial plants to persist for long periods below-ground (Klimešová and Klimeš, 2007; Reintal *et al.*, 2010). In this context, an obvious asymmetry between compartments is expected especially in water-limited ecosystems, where a greater root allocation is the norm (Schenk and Jackson, 2002; Mokany *et al.*, 2006). The ability to detect AM fungal–plant associations might therefore be constrained if only plant above-ground data are used, because they might not accurately depict the below-ground plant community. Recent advances in the use of DNA metabarcoding to determine root diversity (Matesanz *et al.*, 2019; Cabal *et al.*, 2021) offer a means to improve the assessment of the interactions between plant roots and AM fungal communities.

Empirical studies have suggested that the relationships between AM fungi and plants above-ground might differ from those established with roots (e.g. Weigelt *et al.*, 2021; Xia *et al.*, 2021). Plant species vary in their ability to aerate the soil, in the physical changes induced by root growth or in the modifications of temperature and moisture under their canopy (Breshears *et al.*, 1998). For example, AM fungal composition in forest roots can be affected by canopy-mediated light availability (Koorem *et al.*, 2017). Moreover, plants and AM fungi can respond in a similar manner to soil physicochemical properties (Jamiołkowska *et al.*, 2018). This can lead to misleading inferences about the importance of plant community attributes in generating patterns of AM fungal diversity. Conversely, opposite responses of plants and fungi to soil physicochemical properties could mask realized associations between plants and AM fungi. Thus, simultaneous assessment of the combined and independent effects of above- and below-ground plant communities, together with their shared variation with the soil physicochemical properties, on AM fungal diversity is needed to provide a more complete picture of the complex nature of AM associations.

A useful approach that might help to formulate hypotheses related to the abiotic and biotic factors shaping heterogeneous AM fungal distributions is the identification of phylogenetic patterns in AM fungal community composition. The phylogenetic similarity of co-occurring AM fungal taxa has been used previously not only to infer community assembly processes (Liu *et al.*, 2015; Vályi *et al.*, 2016) or to identify key plant determinants of AM fungal diversity (Montesinos-Navarro *et al.*, 2015; Valverde-Barrantes *et al.*, 2016; Chen *et al.*, 2017; López-García *et al.*, 2017), but also to disentangle the existence of co-evolutionary mechanisms, such as exudate release (Brundrett, 2002; Brundrett and Tedersoo, 2018). This phylogenetic approach is based on the idea that closely related species tend to respond to their biotic and abiotic environments in a more similar manner than species selected at random (i.e. they exhibit phylogenetic signal in their ecological interactions). Therefore, species with shared evolutionary history are expected to be ecologically and functionally more similar than more distant relatives (Harvey and Pagel, 1991; Losos, 2008). For example, AM fungi exhibit a strong phylogenetic signal in hyphal growth, root colonization and spore size (Powell *et al.*, 2009; Koch *et al.*, 2017; Aguilar-Trigueros *et al.*, 2019). A recent study showed that the phylogenetic structure of AM fungi can be explained by plant functional traits (López-García *et al.*, 2017), which might also be phylogenetically structured (Valverde-Barrantes *et al.*, 2016; Xia *et al.*, 2021). Furthermore, variations in the abiotic environment, such as soil fertility or pH, can also drive changes in the phylogenetic structure of AM fungal communities (Liu *et al.*, 2015; Davison *et al.*, 2021; Li *et al.*, 2021). Thus, a detailed analysis of the phylogenetic structure of plant and AM fungal communities might shed light on key aspects of these symbiotic relationships interacting with the abiotic environment.

Here, we assess the role of above-ground and below-ground plant community attributes, mainly composition and diversity, and soil physicochemical properties in predicting the spatial variation in the community structure of AM fungi considering the scale at which plant–plant interactions occur in a semiarid Mediterranean scrubland. To achieve this goal, we combine spatially explicit sampling of standing vegetation with next-generation sequencing to unravel the distributions of both roots and AM fungal communities, while accounting for variations in soil physiochemical properties. In water-limited ecosystems, the decoupling between above- and below-ground diversities is exacerbated at fine scales (Schenk and Jackson, 2002; Hiiesalu *et al.*, 2012; Illuminati *et al.*, 2021). Thus, Mediterranean scrublands provide an ideal model in which to test the relative importance of above- and below-ground plant community attributes as drivers of AM fungal communities. In a previous study in the same system, López-Angulo *et al.* (2020) found that the soil generalist fungal diversity and composition were better explained by above-ground plant community attributes compared with below-ground plant community attributes, but information on AM fungal communities and the influence of phylogeny on community assembly is still lacking. Given that above- and below-ground plant communities might drive different processes structuring AM fungi–plant associations, we hypothesize that above- and below-ground plant community attributes will jointly, but also independently, explain patterns of diversity and composition of AM fungal communities. More particularly, we ask whether assessing the more easily accessible above-ground vegetation is a reliable proxy of the linkages between plant roots and AM fungi. In addition, we test the hypothesis that phylogenetically closely related plant species might harbour more closely related AM fungal communities.

## MATERIALS AND METHODS

### Study area and soil sampling

The study was performed in a dry Mediterranean scrubland located 50 km south-east of Madrid in central Spain (40°16ʹ08.5″N, 3°08ʹ11.1″W; 781 m a.s.l.). The mean temperature is 12.8 °C and mean annual precipitation 452 mm, with almost no precipitation during summer. The soil is calcareous and classified as Xeric Calcigypsids (Soil Survey Staff, 2014). The area is densely vegetated by dwarf scrubs dominated by *Thymus vulgaris*, *Bupleurum fruticescens* and *Helianthemum cinereum* interspersed with perennial grasses, such as *Stipa pennata*, *Avenula bromoides* and *Koeleria vallesiana*, and occasionally, with *Quercus coccifera* and *Quercus ilex* subsp. *ballota*.

**Fig. 1.**
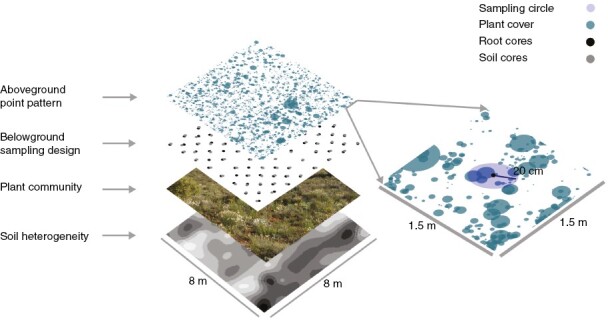
Sampling design. Different layers represent, from bottom to top, the soil heterogeneity, the sampled plant community (64 m^2^), the grid of root and arbuscular mycorrhizal fungal–soil cores, and the point pattern of the above-ground plant community (each point represents an individual, with size proportional to plant mean cover).

We mapped all the above-ground perennial individuals within an 8 m × 8 m plot established in a representative area of the scrubland (Fig. 1) in May 2016. The plot size guaranteed the inclusion of a high number of individuals (8551) and species (45 perennials) belonging to a wide range of plant families (18) (Supplementary Data Table S1). We located each individual plant by recording its rooting point, except for rosette plants, perennial herbs and tussocks, for which rooting points were assigned to the centroid of the plant, using a real-time GPS (Viva GS15; Leica, Wetzlar, Germany; absolute precision 1 cm). For each individual plant, we measured the longest diameter of its crown and the perpendicular diameter to estimate the projection area of the crown. After mapping, we established an 8 × 8 regular grid within the plot, resulting in 64 quadrats (1 m^2^) and set 64 soil sampling points in the centre of each quadrat (Fig. 1). We set 20 additional soil sampling points close to the corners of the plot to increase the spatial resolution at finer scales (Supplementary Data Fig. S1), resulting in 0.70 m spacing between sampling points in these areas (84 soil sampling points in total). Centred around each soil sampling point, we delimited an above-ground sampling circle of 20 cm radius (84 above-ground sampling circles). For each species found within each above-ground sampling circle, we used the sum of all the intersection areas (in centimetres squared) between the projection area of the crowns of the individuals and the sampling circle as an estimation of above-ground species cover. We selected 20 cm because this was the radius that maximized the similarity in diversity between below- and above-ground plant assemblages in the same plant community (Illuminati *et al.*, 2021).

At each soil sampling point, two contiguous soil samples were collected using steel cores (10 cm in depth by 5 cm in diameter). One soil sample was used to evaluate AM fungal diversity and soil physicochemical variables (hereafter, AMF–soil sample), and the other one to assess the below-ground plant community (hereafter, root sample). We sieved and homogenized the soil of the AMF–soil sample through a 2 mm mesh. We stored a 0.1 g subsample of each homogenized sample for molecular analyses of AM fungal diversity (see below in Arbuscular mycorrhizal fungal characterization section) and a 50 g subsample of air-dried soil for soil physicochemical analyses. Specifically, we determined four variables related to nutrient stocks (organic carbon, total nitrogen, available phosphorus and potassium), two dynamic variables related to the soil microbial activity (acid phosphatase and β-glucosidase enzymatic activities) and several physical variables (pH, electrical conductivity, sand, silt and clay contents; for summary statistics, see Supplementary Data Table S2). In parallel, all plant roots from the root samples were filtered through a 1 mm mesh, thoroughly washed within 48 h after field collection and then centrifuged at 3000*g* for 30 s to remove excess water. We weighed the fresh root biomass and homogenized it by cutting roots into small pieces. We stored 0.1 g of root biomass per sample at −80 °C for subsequent DNA metabarcoding.

### Arbuscular mycorrhizal fungal characterization

#### DNA isolation

DNA from the AMF–soil samples was isolated using the DNeasy PowerSoil isolation kit (Qiagen, CA, USA) following the manufacturer’s protocol. An extraction blank was included in each of the seven DNA extraction rounds to check for cross-contamination.

#### DNA metabarcoding library preparation and sequencing

For library preparation, Glomeromycota 18S (SSU) rRNA gene sequences were amplified from DNA extracts using the primers NS31 (5ʹ-TTGGAGGGCAAGTCTGGTGCC-3ʹ) and AML2 (5ʹ-GAACCCAAACACTTTGGTTTCC-3ʹ) (Simon *et al.*, 1992; Lee *et al.*, 2008). PCRs were performed in a total volume of 25 µL, containing 0.5 µm of the primers, 12.5 µL of Supreme NZYTaq 2× Green Master Mix (NZYTech, Lisbon, Portugal), 1–2.5 µL of template DNA and ultrapure water up to 25 µL. After the first PCR (95 °C for 5 min, followed by 25–35 cycles of 95 °C for 30 s, 47–50 °C for 30 s and 72 °C for 30 s, with a final extension at 72 °C for 10 min), a second PCR, with five cycles and 60 °C as the annealing temperature, was performed to attach the index sequences required for multiplexing different libraries in the same sequencing pool. Negative controls with no DNA were included to check for contamination during library preparation. Libraries were run on 2 % agarose gels stained with GreenSafe (NZYTech, Lisbon, Portugal), viewed under ultraviolet light to verify the library size and purified using Mag-Bind RXNPure Plus magnetic beads (Omega Biotek, Norcross, GA, USA). Libraries were then pooled in equimolar amounts according to quantification provided by the Qubit dsDNA HS Assay Kit (Thermo Fisher Scientific, Waltham, MA, USA). The pool was sequenced in a MiSeq PE300 v3 run at Unidad de Genómica (Fundación Parque Científico de Madrid, Spain).

#### Bioinformatic analyses

The quality of the Illumina paired-end raw FASTQ files was checked using FastQC (Andrews, 2010). Paired-end assembly of the R1 and R2 reads was performed with FLASH (Magoč and Salzberg, 2011). Sequences were labelled (demultiplexed) using the script ‘multiple_split_libraries.py’ implemented in QIIME (Caporaso *et al.*, 2010), while setting a minimum Phred quality score of 20. The FASTA files were processed using the VSEARCH bioinformatic tool (Rognes *et al.*, 2016). Sequences were dereplicated (-derep fulllength), clustered at a similarity threshold of 100 % (-cluster fast,-centroids option), and sorted (-sortbysize). *De novo* chimeras were removed using the UCHIME algorithm (Edgar *et al.*, 2011) implemented in VSEARCH. Then, sequences were assigned to an operational taxonomic unit (OTU) (-search global).

#### Taxonomic assignment

We performed taxonomic assignment of the AM fungal OTU by querying the clustered centroids against the Maarj*AM* reference database (Öpik *et al.*, 2010; accessed 3 October 2018), using the script ‘assign_taxonomy.py’ implemented in Qiime and the BLAST algorithm with a maximum E-value of 1 × 10^−50^ and a minimum percentage identity of 90 % in order to assign the Glomeromycota sequences to an OUT (Morgan and Egerton-Warburton, 2017). An OTU table with the number of sequences of each OTU in each sample was created. OTU tables were subjected to quality filtering to remove OTUs with low frequency in the whole dataset (0.005 %; Bokulich *et al.*, 2013) and low abundance (0.1 % threshold; Esling *et al.*, 2015). Ten samples with no sequences and one sample with four sequences after all filtering steps were removed from further analyses.

#### Phylogenetic tree

We built the AM fungal phylogeny from the representative sequences of each OTU. We calculated a maximum likelihood (ML) phylogeny using the GTR+I+G nucleotide substitution model [selected on the basis of corrected Akaike information criterion (AICc); ModelTest; Schliep, 2011] and 100 fast bootstrap replicates.

### Root characterization

Root sequencing was performed following the pipeline described above, with exceptions specific to the group (for more details, see Matesanz *et al.*, 2019; Illuminati *et al.*, 2021). Specifically, a fragment of the *rbcL* chloroplast gene sequence (550 bp) was amplified using the primers rbcLa-F (5ʹ-ATGTCACCACAAACAGAGACTAAAGC3-3ʹ) and rbcLa-R (5ʹ-GTAAAATCAAGTCCACCRCG-3ʹ) (Levin *et al.*, 2003; Kress *et al.*, 2009). After sequencing on the Illumina MiSeq PE300 v.3 run, we used VSEARCH to confirm species assignments (99 % match to reference) using an in-house reference database containing the *rbcL* sequences of all plant species sequenced individually (see sequences in the Supplementary Data Sequences File S1). Four very closely related species pairs (*Stipa pennata* and *Stipa tenacissima*; *Teucrium capitatum* and *Teucrium gnaphalodes*; *Thymus vulgaris* and *Thymus lacaitae*; and *Quercus coccifera* and *Q. ilex*) were grouped at the genus level because their *rbcL* sequences were identical. Below-ground species abundance was estimated as the number of reads for each species after rarefaction of each plant community (1768 sequences per sample) to equal sequence numbers. We reconstructed the *rbcL* phylogenetic tree from each representative sequence. We used the in-house reference database to build an ML phylogenetic tree using the GTR+I+G nucleotide substitution model and 100 fast bootstrap replicates.

### Estimation of AM fungal and plant community attributes

#### Arbuscular mycorrhizal fungal taxonomic and phylogenetic composition and diversity

Arbuscular mycorrhizal fungal taxonomic composition was assessed from the AM fungal abundance matrix. AM fungal phylogenetic composition was computed as the AM fungal phylogenetically weighted species composition matrix (Pillar and Duarte, 2010), by weighting the AM fungal abundances by their phylogenetic relationships estimated from the phylogenetic tree (Supplementary Data Methods S1; Debastiani & Pillar, 2012). AM fungal taxonomic diversity was estimated as the taxon richness in each sample (i.e. total number of OTUs). Before estimating AM fungal taxonomic diversity, AM fungal sequence reads were rarefied to the minimum number of sequences in a soil sample (1451 sequences per sample; Supplementary Data Fig. S2; Methods S1), to account for the unequal number of sequences among samples. AM fungal taxonomic diversity based on rarefied data was highly correlated with unrarefied AM fungal taxonomic diversity (*r* = 0.998). AM fungal phylogenetic diversity was estimated as Rao’s quadratic entropy index (Rao, 1982), and one outlier was removed from subsequent statistical analysis.

#### Above-ground plant taxonomic composition and diversity

Two measures of above-ground plant taxonomic composition were calculated as the scores of each above-ground plant sample on the first two axes of a non-metric multidimensional scaling ordination (nMDS; Supplementary Data Methods S1). nMDS is a robust method to reduce the dimensionality of the AM fungal communities in two or three variation axes (Legendre and Legendre, 2012). We performed the nMDS using the Bray–Curtis distance, based on the Hellinger-transformed plant cover data from each sampling circle (Barberán *et al.*, 2015). The stress value (i.e. the parameter that shows the goodness of fit of the ordination) was 0.19. The first axis (above-ground taxonomic composition.1) captured a gradient from above-ground plant samples dominated by the cover of species such as *Jurinea humilis*, *Helianthemum syriacum* and *Phlomis lychnitis* (negative values; Supplementary Data Fig. S3A) to samples dominated by *Fumana thymifolia*, *Teucrium gnaphalodes* and *Ononis tridentata*. The negative values from the second axis (above-ground taxonomic composition.2) were related to *Quercus rotundifolia*, *Salvia lavandulifolia* and *Sideritis hirsuta*, whereas the positive values were related to *Santolina chamaecyparissus*, *Linum narbonense* and *Cephalaria leucantha* (Supplementary Data Fig. S3A). Above-ground plant taxonomic diversity was estimated as the plant species richness (i.e. total number of species) in each above-ground plant circle with radius 20 cm.

#### Above-ground plant phylogenetic composition and diversity

To estimate above-ground plant phylogenetic composition, we first computed a matrix of the above-ground plant phylogenetically weighted species composition (Pillar and Duarte, 2010), using the above-ground species data matrix and the plant phylogenetic tree. Then, for each sample, two measures of plant phylogenetic composition were calculated as the scores of each sample on the first two axes of an nMDS using the Bray–Curtis distance on the Hellinger-transformed matrix. The stress value for the nMDS was 0.12. Unlike the first axis of the nMDS conducted on above-ground plant taxonomic composition, in which species belonging to the same family could be found at both ends of the taxonomic compositional gradient (e.g. *Teucrium gnaphalodes* and *Phlomis lychnitis*; Supplementary Data Fig. S3A), members of the same family are found at the same end of a phylogenetic compositional axis. The negative values of the first phylogenetic composition axis (above-ground phylogenetic composition.1) were related to grasses (Poaceae), whereas all other families (mainly forbs) were grouped towards the positive values (Supplementary Data Fig. S3B). The negative values of the second phylogenetic composition axis (above-ground phylogenetic composition.2) were related to Lamiaceae species, whereas the positive values were related to Cistaceae and Fabaceae species (Supplementary Data Fig. S3B). The above-ground phylogenetic diversity was estimated using the Rao index.

#### Below-ground plant taxonomic and phylogenetic composition and diversity

Taxonomic and phylogenetic composition and diversity were computed as for the above-ground community but using the number of sequences per root sample instead of plant cover.

The two below-ground plant taxonomic composition axes were negatively related to assemblages dominated by roots of *Ononis tridentata*, *Staehelina dubia* and *Lithodora fruticosa* (below-ground taxonomic composition.1) and of *Phlomis lychnitis*, *Hippocrepis commutata* and *Lithodora fruticosa* (below-ground taxonomic composition.2). Furthermore, they were positively related to assemblages dominated by roots of *Linum narbonense*, *Euphorbia nicaeensis* and *Fumana thymifolia* (below-ground taxonomic composition.1) and of *Euphorbia nicaeensis*, *Linum narbonense* and *Salvia lavandulifolia* (below-ground taxonomic composition.2) (Supplementary Data Fig. S3C). The first below-ground phylogenetic composition axis was negatively related to Cistaceae and Lamiaceae species and positively related to Poaceae species. The second below-ground phylogenetic composition axis was negatively related to Poaceae species and positively related to Fabaceae species (Supplementary Data Fig. S3D). The nMDS for the taxonomic and phylogenetic below-ground composition had stress values of 0.21 and 0.07, respectively. Below-ground plant taxonomic and phylogenetic diversities were estimated as plant species richness (i.e. total number of taxa) and the Rao index, respectively, in each sample.

#### Above-ground plant cover and root biomass

Above-ground plant cover was estimated as the sum of the cover of all species within the circle with a radius of 20 cm, and root biomass was assessed as the total root biomass per root sample.

#### Soil and spatial covariates

To reduce the number of soil physicochemical variables, we performed a principal components analysis (PCA) with varimax rotation. Before performing the PCA, we estimated the best number of components to retain in our analyses using the smoothing approximation of the cross-validation criterion implemented by Josse and Husson (2012). We retained the first four principal components. The first axis, explaining 22 % of the variance, was highly related to soil texture, varying from soil with high sand content at one end of the axis to soil with high clay and slit content at the other. The second axis (explaining 19 %) was mainly related to variations in soil organic carbon. The third axis (explaining 18 %) was positively correlated with nitrogen and phosphorus content (fertility). Finally, the fourth axis (explaining 16 %) was associated with variations in pH and conductivity (salinity) (for more details, see table S2 in the paper by López-Angulo *et al.*, 2020).

To account for unmeasured spatially structured variables (i.e. spatial covariates), we generated a set of Moran’s eigenvectors from the coordinates of each sampling point using distance-based Moran’s eigenvector maps (dbMEM; Legendre and Legendre, 2012). Initially, we fitted a trend surface (i.e. a linear regression on the *x*- and *y*-coordinates) to remove spatial trends in the four response variables (i.e. AM fungal taxonomic and phylogenetic diversity, taxonomic and phylogenetic composition). The residuals of these regressions were used to compute dbMEM eigenvectors (Borcard and Legendre, 2002). To select the dbMEM eigenvectors that significantly contributed to explain AM fungal diversity and composition, we performed a forward selection procedure with double-stopping criterion (α = 0.05, 9999 permutations; Blanchet *et al.*, 2008) of the dbMEM eigenvectors. We repeated this procedure independently for each dependent variable (i.e. the AM fungal taxonomic and phylogenetic diversity and composition), such that a different number of dbMEM variables was selected in each case (AM fungal taxonomic diversity = 5; phylogenetic diversity = 3; taxonomic composition = 2; phylogenetic composition = 3). The *x*- and *y*-coordinates were also included as predictors in the final models.

### Statistical analyses

To determine whether above-ground plant community attributes were correlated significantly with the plant community attributes estimated below-ground, Pearson correlations were conducted. We included all plant community attributes as predictors because correlation coefficients varied between 0.5 and −0.5 (Supplementary Data Figs S4 and S5). All these statistical analyses, and the subsequent ones, were performed in R (v.4.0.2; R Core Team), and a detailed description of packages used can be found in Supplementary Data Methods S1.

#### Arbuscular mycorrhizal fungal composition

We used partial redundancy analysis (pRDA; Legendre *et al.*, 2012) to study the above- and below-ground effects of the plant community on the variation of AM fungal composition. As the response matrix, we used either the taxonomic or the phylogenetically weighted AM fungal composition matrices. As predictors, we used the above- and below-ground plant diversity and composition, root biomass and above-ground plant cover. The effect of the below-ground plant diversity and composition was tested after accounting for the effects of the above-ground plant diversity and composition, and vice versa, and root biomass and above-ground plant cover. To account for soil effects and residual spatial variation, we constrained the ordinations by soil physicochemical properties (PCAs) and spatial covariates (dbMEM eigenvectors). To avoid multicollinearity, we performed independent pRDAs for the taxonomic and phylogenetic plant community predictors. We conducted the pRDAs with forward selection using the double-stopping criteria (*P* < 0.05 and adjusted *R*^2^ < global *R*^2^; Blanchet *et al.*, 2008). We tested the significance of predictors using the Monte Carlo test based on 999 permutations. Before statistical analyses, we applied a Hellinger transformation on the AM fungal and plant community matrices (Legendre and Gallagher, 2001). We applied a square root transformation on root biomass and the salinity gradient (second PCA axis of the soil PCA).

#### Arbuscular mycorrhizal fungal diversity

We fitted generalized linear models to both AM fungal taxonomic and phylogenetic diversity to assess the effects of the plant community attributes (above- and below-ground taxonomic and phylogenetic composition and diversity, root biomass and above-ground plant cover), controlling the effects of soil physicochemical properties (PCAs) and spatial covariates (dbMEM eigenvectors). The response of the AM fungal taxonomic diversity to predictors was evaluated considering a Poisson error distribution and logarithmic link function, whereas the response of AM fungal phylogenetic diversity was analysed using a Gaussian error distribution and identity link function. We used a model selection procedure based on minimizing the AICc to select the best predictors of AM fungal taxonomic and phylogenetic diversities. The models were ranked according to the AICc. We calculated model-averaged parameter estimates over the set of models with ∆AICc < 2 (Burnham and Anderson, 2002). We estimated 95 % confidence intervals (CIs) around model-averaged parameter estimates, considering a parameter to be significant if the 95 % CI excluded zero (Burnham and Anderson, 2002). We checked the absence of multicollinearity in all models using the variance inflation factor. In all cases, variance inflation factors were smaller than four, indicating absence of collinearity (Zuur *et al.*, 2010). To avoid overfitting, we allowed a maximum of seven predictors in the candidate models because it is recommended that the sample size (i.e. number of soil cores) should be ten times greater than the maximum number of predictors (Harrell *et al.*, 1984; Hair *et al.*, 2014; Supplementary Data Methods S1).

## RESULTS

### Taxonomic description of the AM fungal and plant communities

A total of 359 507 18S rRNA gene sequence reads were assigned to 1267 AM fungal OTUs, ranging from 1451 to 13 931 reads per sample. AM fungal communities were dominated by taxa within the Glomeraceae family, representing 91 % of the sequences (Supplementary Data Fig. S6). The remaining OTUs belonged to the Acaulosporaceae, Ambisporaceae, Archaeosporaceae, Claroideoglomeraceae, Diversisporaceae, Gigasporaceae and Paraglomeraceae (Supplementary Data Table S3). AM fungal taxonomic diversity ranged from 8 to 130 OTUs per sample (62.9 ± 22.4 OTUs; mean ± SD).

A total of 30 plant taxa, 26 identified at the species level and 4 at the genus level, were found below-ground across all soil samples (identified through DNA metabarcoding), whereas 39 plant species were detected above-ground in the area corresponding to all circles of 20 cm radius. The below- and above-ground taxonomic diversity ranged from 3 to 12 plant species (7 ± 2; mean ± SD) and from 3 to 16 (8 ± 2.7; mean ± SD), respectively. The above-ground and below-ground plant composition were significantly correlated, both taxonomically (Supplementary Data Fig. S4) and phylogenetically (Supplementary Data Fig. S5). The above-ground plant taxonomic diversity was weakly and significantly correlated with below-ground plant taxonomic diversity (*r* = 0.36, *P* < 0.01). In contrast, the above-ground plant phylogenetic diversity was not correlated with below-ground plant phylogenetic diversity (Supplementary Data Fig. S5).

### Arbuscular mycorrhizal fungal community composition

Partial pRDAs showed that the forward-selected above- and below-ground attributes of plant communities together explained 1.2 and 5.6 % of the total variance of taxonomic and phylogenetic AM fungal composition, respectively (for forward-selected predictors, see Table 1; Supplementary Data Fig. S7), and the soil physicochemical properties (soil fertility) explained 0.6 % of the taxonomic AM fungal composition (Table 1; Supplementary Data Fig. S7). We found that below-ground plant taxonomic composition and above-ground plant phylogenetic composition explained unique fractions of variation in the AM fungal taxonomic and phylogenetic composition (Table 1; Supplementary Data Fig. S8). Specifically, Glomeraceae taxa were associated with forbs, whereas the rest of the families tended to be associated with grasses (Supplementary Data Fig. S9). Above-ground taxonomic plant diversity, but not below-ground plant diversity, was also associated with variations of AM fungal taxonomic composition (Table 1). This indicates that more species-rich plant assemblages above-ground led to significant changes in the identity of the taxa co-occurring in the AM fungal communities.

**Table 1.** Significance level (*P*-values) and adjusted coefficient of determination (adjusted *R*^2^) of the effects of above- and below-ground plant attributes (taxonomic and phylogenetic diversity and composition) and soil physicochemical properties (pH and fertility) on the arbuscular mycorrhizal fungal taxonomic and phylogenetic composition and diversity.

	Arbuscular mycorrhizal fungal composition				Arbuscular mycorrhizal fungal diversity			
	Taxonomic		Phylogenetic		Taxonomic		Phylogenetic	
Plant predictor set	Adjusted *R*^2^	*P*-value	Adjusted *R*^2^	*P*-value	Adjusted *R*^2^	*P*-value	Adjusted *R*^2^	*P*-value
Taxonomic above-ground	–	–	–	–	–	–	–	–
Diversity	2.1	0.004	–	–	–	–	–	–
Composition.1	–	–	–	–	4.6	<0.001	–	–
Taxonomic below-ground	–	–	–	–	–	–	–	–
Composition.1	1.9	0.029	4.8	0.019	1.8	<0.001	5.0	0.021
Phylogenetic above-ground	–	–	–	–	–	–	–	–
Diversity	1.9	0.007	–	–	6.8	<0.001	–	–
Composition.1	2.0	0.007	4.1	0.029	2.3	<0.001	3.4	0.025
Composition.2	–	–	–	–	4.4	<0.001	5.5	0.005
Phylogenetic below-ground	–	–	–	–	–	–	–	–
Composition.2	1.9	0.025	–	–	–		–	–
Soil physicochemical properties	–	–	–	–	–	–	–	–
pH	–	–	–	0.012	–	–	5.0	0.018
Fertility	–	0.035	–	–	1.7	<0.001	–	–

The significance of predictors on the AM fungal composition was tested against 999 Monte Carlo permutations in partial redundancy analyses. The significance of predictors on the AM fungal diversity was tested using a *z*-statistic after estimating 95 % confidence intervals around model-averaged parameter estimates using generalized linear models. The reported adjusted *R*^2^ represents the unique variance explained by each variable after variance partitioning (see shared variance in Supplementary Data Figs S7 and S8). Only variables significant in at least one model are shown in the table. Composition.1 and composition.2 are variables calculated as the scores of each sample on the two first axes of non-metric multidimensional scaling ordinations (nMDS).

The AM fungal composition was also associated with variations in soil physicochemical properties (Table 1). Specifically, pRDA biplots showed that most AM fungal taxa belonging to Diversisporaceae and Claroideoglomeraceae families were associated with poorer soils, and the taxa belonging to Glomeraceae were associated with alkaline soils (pH ≈ 8; Supplementary Data Fig. S10). pRDA ordinations also showed that the AM fungal communities were spatially structured (Supplementary Data Table S4).

### Arbuscular mycorrhizal fungal diversity

**Fig. 2.**
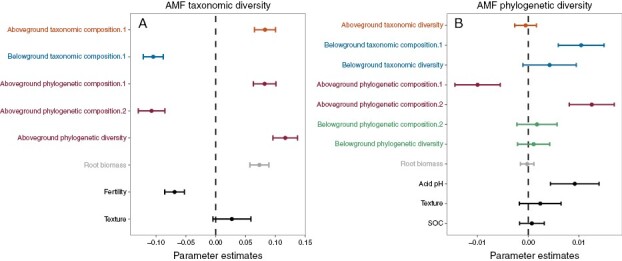
Effects of the above- and below-ground taxonomic and phylogenetic plant community attributes and soil physicochemical properties on the arbuscular mycorrhizal fungal (AMF) taxonomic diversity (A) and phylogenetic diversity (B). The averaged parameter estimates (standardized regression coefficients) of model predictors and the associated 95 % confidence intervals are shown. The effects of taxonomic and phylogenetic plant community attributes were evaluated in separate models (for model selection, see Supplementary Data Table S5). Only those predictors selected by the AICc are shown. Note that composition.1 and composition.2 are variables calculated as the scores of each sample on the two first axes of non-metric multidimensional scaling ordinations (Supplementary Data Fig. S3). Abbreviations: SOC, soil organic carbon.

Arbuscular mycorrhizal fungal taxonomic diversity was positively associated with above-ground plant phylogenetic diversity (Fig. 2), and overall, this was consistent for all fungal families (Supplementary Data Fig. S11). AM fungal taxonomic diversity was also associated with variations in above- and below-ground plant taxonomic composition (Fig. 2), indicating that different combinations of plant species can lead to richer (higher number of OTUs) AM fungal communities. Furthermore, the two nMDS axes of above-ground phylogenetic plant composition affected the AM fungal taxonomic diversity, and, in the opposite direction, the AM fungal phylogenetic diversity (Fig. 2). This result indicated that areas with above-ground cover of Lamiaceae species were related to more taxa-rich but less phylogenetically diverse AM fungal communities, matching the increase in Glomeraceae taxa (Supplementary Data Fig. S11). The abundance of Poaceae, Fabaceae and Cistaceae species led to poor (in terms of the number of OTUs) but more phylogenetically diverse AM fungal communities (Fig. 2). This relationship was probably attributable to the finding that these plant groups decreased Glomeraceae taxa and increased Claroideoglomeraceae and Paraglomeraceae (Supplementary Data Fig. S11).

The AM fungal taxonomic diversity decreased with soil fertility (1.1 % of explained variance), whereas AM fungal phylogenetic diversity decreased with soil alkaline pH (7.2 % of explained variance; Supplementary Data Fig. S12). The spatial eigenvectors representing unmeasured spatially structured factors exerted positive effects on AM fungal taxonomic diversity and negative effects on AM fungal phylogenetic diversity (Supplementary Data Fig. S12).

## DISCUSSION

Our findings provide evidence of a heterogeneous distribution of AM fungal communities at the spatial scale in which plants interact in a Mediterranean scrubland. We found that the plant community attributes, soil physicochemical properties and spatial variables jointly explained a significant fraction of the local distribution of AM fungal communities. Thus, we demonstrated that using sets of different predictors related to above- and below-ground vegetation, soil and space provided a more complete picture of the complex nature of AM associations. Several studies have also shown a high spatial variation of the AM fungal communities at fine scales (Horn *et al.*, 2017; Avio *et al.*, 2020). However, unlike these studies, we found that plant community attributes predicted unique fractions of AM fungal community structure and composition. Importantly, these results point out that above- and below-ground plant community attributes that predicted unique fractions of AM fungal community structure were decoupled, as previously shown in the same plant community (Illuminati *et al.*, 2021) and in other environments (Schenk and Jackson, 2002; Reintal *et al.*, 2010; Hiiesalu *et al.*, 2012). This above-ground plant influence, together with a positive effect of root biomass and negative effect of soil fertility on AM fungal taxonomic diversity, has also been documented recently for the taxonomic diversity of key soil fungi in the same study system (López-Angulo *et al.*, 2020), suggesting that fungi belonging to subphylum Glomeromycotina respond in a similar manner to general ecological drivers of soil fungal diversity (i.e. nutrient gradient and habitat availability) (Legay *et al.*, 2014). Nevertheless, AM fungal diversity and composition were also more directly associated with the below-ground compartment of the plant community (particularly with the below-ground plant composition) than soil fungal diversity and composition (López-Angulo *et al.*, 2020), confirming the expected direct relationship between AM fungi and plant roots.

Many studies to date have used above-ground information to establish relationships between plant and AM fungal communities, obviating the root fraction and thus, assuming a certain symmetry between both community compartments (Horn *et al.*, 2017; Van Geel *et al.*, 2018; Bittebiere *et al.*, 2020). The recent development of high-throughput DNA metabarcoding has offered a means to determine root diversity (Matesanz *et al.*, 2019; Cabal *et al.*, 2021), providing opportunities for a better and more realistic understanding of the interactions between plants and AM fungi. Despite these methodological advances to identify plant species below-ground, our results show that above-ground information such as plant cover, which is easy to estimate visually, might also be an indicator of the linkages between plants and AM fungi. The rationale behind the strong association between above-ground plant diversity and these obligate root symbionts might be that the larger sampling size above-ground provides an extensive characterization of the entire plant community, including that below-ground. On the contrary, soil core sampling might fail to capture all species recorded in the above-ground sampling rings. Although the radius for the above-ground sampling circles maximizes the similarity between both plant compartments, significant discrepancies remain between them in this community (Illuminati *et al.*, 2021). For example, plant shoot and root systems can exhibit large differences in lateral spread and temporal turnover dynamics (Schenk and Jackson, 2002; Sun *et al.*, 2016). In this sense, our above-ground sampling might have provided a longer-lasting picture of the plant community, because above-ground effects might be more stable temporally than those below-ground, given that root distributions change faster over time than the above-ground parts (Peek *et al.*, 2005; Sun *et al.*, 2016). These different temporal dynamics could lead to AM fungal communities being coupled differently with both compartments of plant communities.

The AM fungal diversity and composition in our Mediterranean scrubland were explained mainly by variations in the above-ground plant composition, showing that certain plant species can stimulate the performance of particular AM fungal taxa. These findings, which denote some degree of preferential association between plants and AM fungi, concur with results of microcosms and observational studies conducted in other systems (Johnson *et al.*, 2004; Hausmann and Hawkes, 2009; Alguacil *et al.*, 2019; Šmilauer *et al.*, 2020). Furthermore, the fact that plant composition, but not plant diversity, affected AM fungal taxonomic diversity suggests that the identity of plant species influences the assembly of AM fungal communities more directly than the variety or the number of plant species (Trinder *et al.*, 2009; Neuenkamp *et al.*, 2021). Another explanation for the lack of relationship between below-ground plant and AM fungal taxonomic diversity would be the occurrence of common mycorrhizal networks (Van Der Heijden and Horton, 2009; van der Heijden *et al.*, 2015). In these networks, few AM fungal taxa colonize different plant roots, interconnecting different plant species and thus leading to a decoupling between AM fungal and plant diversity, especially at this fine scale.

When the phylogenetic relationships were considered in the analyses, above-ground plant phylogenetic composition (first axis of nMDS), which separated grasses (Poaceae) from forbs (Supplementary Data Fig. S3B), was the only predictor explaining a significant and unique fraction of variation in the AM fungal phylogenetic composition and diversity (Supplementary Data Fig. S8). This result is in line with other studies showing the existence of a phylogenetic signal for AM associations (Yang *et al.*, 2012; Montesinos-Navarro *et al.*, 2015; Chen *et al.*, 2017; López-García *et al.*, 2017). Our findings indicated that AM fungal taxa belonging to family Glomeraceae tended to be associated with forbs, whereas other AM fungal families (especially Paraglomeraceae, Ambisporaceae, Claroideoglomeraceae and Archaeosporaceae) were more related to grasses. This was probably the reason why AM fungal communities related to grasses were more phylogenetically diverse although they exhibited a lower number of OTUs. This agrees with Šmilauer *et al.* (2020), who found that plant species (mainly Poaceae) negatively affecting the richness of the AM fungal communities also positively affected the AM fungal phylogenetic diversity (mean nearest taxon phylogenetic distance).

In addition, our linear models showed that Lamiaceae species were positively associated with a larger number of OTUs dominated by Glomeraceae taxa, whereas Fabaceae and Cistaceae species supported a larger phylogenetic diversity of AM fungal communities. These results could be explained by the relative fitness differences and the niche differences among competing AM fungal taxa, as postulated by the coexistence theory (Chesson, 2000). Plant assemblages dominated by Lamiaceae, the most diverse and abundant plant family in our scrubland, led to more phylogenetically clustered AM fungal communities but with a larger number of OTUs, dominated by Glomeraceae taxa. This pattern might be the result of asymmetric competition (Mayfield and Levine, 2010) through the exclusion of poor competitors belonging to distantly related AM fungal lineages produced by Glomeraceae taxa. Members of the Glomeraceae establish a faster (Hart and Reader, 2002), more extensive (Maherali and Klironomos, 2007) and more permanent (Hart and Reader, 2002; López-García *et al.*, 2014) mycelium in roots compared with other AM fungal families, and they might also exhibit higher competitive ability for carbon uptake (Yang *et al.*, 2014). In contrast, in soils dominated by grasses and legumes, niche differentiation might favour AM fungal communities composed of more distantly related taxa, in turn enhancing coexistence among AM fungi. This is supported by the fact that grasses are expected to be less dependent on mycorrhizal symbiosis owing to the high efficiency of their fibrous roots to absorb nutrients (Javaid, 2008). Accordingly, legumes might be associated with phylogenetically overdispersed AM fungal communities because the functional traits needed to associate with N-fixing plants are not phylogenetically conserved (López-García *et al.*, 2017). These opposite patterns of phylogenetic and taxonomic diversity suggesting competitive exclusion were also found in a study of an alpine meadow, in which an increase in soil fertility (N + P) caused a loss of taxonomic diversity (particularly of *Glomus* species), but an increase in genus taxonomic diversity (Liu *et al.*, 2015). Our findings highlight that changes in environmental conditions, including those mediated by specific groups of plants, can shape the outcome of the direct biotic interactions among AM fungi (Thonar *et al.*, 2014; Knegt *et al.*, 2016).

Finally, in our study, the variance of AM fungal phylogenetic diversity and composition explained by the spatial eigenvectors was higher than the variance explained by the plant attributes and soil physicochemical properties. This suggests that the AM fungal assemblage exhibited strong phylogenetic and spatial structuring. Other spatially autocorrelated abiotic factors, together with biotic interactions among soil microorganisms, could be mechanisms that drive the spatial patterns in the phylogenetic structure of AM fungal communities (Maherali and Klironomos, 2007; Knegt *et al.*, 2016). Furthermore, the large unexplained variation of AM fungal diversity and composition observed in our models probably reflects unmeasured deterministic or neutral processes that are not spatially structured at the spatial scales considered in our sampling. Such unexplained variation might include the effect of topographic or microclimatic environmental variations, such as soil moisture and temperature heterogeneity (Kivlin *et al.*, 2011; Teste *et al.*, 2020). Therefore, the inclusion of other abiotic variables that might influence AM fungi and plants in parallel could increase the shared variation between plants and the soil environment.

### Conclusions

We provide evidence that the plant community attributes, soil physicochemical properties and spatial variables jointly affect the fine-scale distribution of AM fungal communities in a Mediterranean scrubland. Importantly, our results highlight that, despite the decoupling found between the above-ground and below-ground plant compartments, the more easily accessible above-ground vegetation might serve as an indicator of the active interactions of AM fungi with plant roots. However, agreeing with our hypothesis, both below- and above-ground plant community attributes also explained unique fractions of the variation in AM fungal composition and diversity at the fine scale in which plants interact. Thus, we emphasize the importance of considering the above-ground plant distribution and soil environmental factors, together with data from the below-ground plant compartment, because they clearly improve our ability to predict the relationships between AM fungal and plant communities. As hypothesized, we show the importance of incorporating the phylogenetic relatedness of mutualistic partners into metrics of diversity and composition to improve our understanding of the ecological and evolutionary mechanisms underlying the assembly of AM fungal communities.

## SUPPLEMENTARY DATA

Supplementary data are available online at https://academic.oup.com/aob and consist of the following.

Sequences File S1: species in-house reference database containing the *rbcL* sequences of all plant species individually sequenced. Methods S1: R packages used for specific applications. Figure S1: core sampling design. Figure S2: rarefaction curve of arbuscular mycorrhizal fungal communities for each soil sample. Figure S3: non-metric multidimensional scaling ordinations showing patterns of variation in plant above-ground and below-ground taxonomic and phylogenetic composition. Figure S4: pairwise scatterplots of the plant community taxonomic attributes. Figure S5: pairwise scatterplots of the plant community phylogenetic attributes. Figure S6: proportion of sequence reads of arbuscular mycorrhizal fungi at the family level. Figure S7: Venn diagrams showing variance partitioning results of AM fungal taxonomic and phylogenetic composition, and taxonomic and phylogenetic diversity explained by plant community attributes, soil properties and spatial covariates. Figure S8: Venn diagrams showing variance partitioning results of AM fungal taxonomic and phylogenetic composition, and taxonomic and phylogenetic diversity explained by the below-ground and above-ground taxonomic and phylogenetic plant community attributes. Figure S9: redundancy analysis biplots showing the relationship of AM fungal taxonomic composition and phylogenetic composition to the below-ground and above-ground taxonomic and phylogenetic plant community attributes. Figure S10: redundancy analysis biplots showing the relationship of AM fungal taxonomic composition and phylogenetic composition to the soil properties and spatial covariates. Figure S11: linear relationships between the AM fungal taxonomic and the taxonomic and phylogenetic plant community attributes. Figure S12: effect of soil physicochemical properties and the spatial covariates. Table S1: list of plant species found in our study plot. Table S2: summary statistics for soil physicochemical properties. Table S3: list of arbuscular mycorrhizal fungi virtual taxa indicating taxonomic ranks, included known species and unidentified cultures. Table S4: ANOVA-like results based on redundancy analysis testing the effect of the forward-selected soil physicochemical properties and spatial covariates on the arbuscular mycorrhizal fungi taxonomic and phylogenetic composition. Table S5: results of the AICc-based model selection based on linear models testing the response of arbuscular mycorrhizal fungi diversity to plant community attributes controlling for the effects of above-ground plant cover, root biomass and soil physicochemical properties.

mcad050_suppl_Supplementary_Material

mcad050_suppl_Supplementary_Data

## Data Availability

Data associated with this paper were deposited in Figshare at: https://figshare.com/s/c9a23084a601b5e42e67. Illumina next-generation DNA sequences were deposited in the NCBI Sequence Read Archive (BioProject accession no. PRJNA638529; www.ncbi.nlm.nih.gov/bioproject/PRJNA638529).

## References

[CIT0001] Aguiar MR , Sala OE. 1999. Patch structure, dynamics and implications for the functioning of arid ecosystems. Trends in Ecology & Evolution 14: 273–277. doi:10.1016/s0169-5347(99)01612-2. 10370263

[CIT0002] Aguilar-Trigueros CA , Hempel S, Powell JR, Cornwell WK, Rillig MC. 2019. Bridging reproductive and microbial ecology: a case study in arbuscular mycorrhizal fungi. The ISME Journal 13: 873–884. doi:10.1038/s41396-018-0314-7. 30504896 PMC6461870

[CIT0003] Alguacil MM , Díaz G, Torres P, Rodríguez-Caballero G, Roldán A. 2019. Host identity and functional traits determine the community composition of the arbuscular mycorrhizal fungi in facultative epiphytic plant species. Fungal Ecology 39: 307–315. doi:10.1016/j.funeco.2019.02.002.

[CIT0004] Allen MF. 2007. Mycorrhizal fungi: highways for water and nutrients in arid soils. Vadose Zone Journal 6: 291–297. doi:10.2136/vzj2006.0068.

[CIT0005] Andrews S. 2010. FastQC: a quality control tool for high throughput sequence data. http://www.bioinformatics.babraham.ac.uk/projects/fastqc

[CIT0006] Avio L , Njeru EM, Oehl F, et al. 2020. Small-scale soil heterogeneity affects the distribution of arbuscular mycorrhizal fungal species in a hot-spot field in a Mediterranean site. Applied Soil Ecology 154: 103631. doi:10.1016/j.apsoil.2020.103631.

[CIT0007] Barberán A , McGuire KL, Wolf JA, et al. 2015. Relating belowground microbial composition to the taxonomic, phylogenetic, and functional trait distributions of trees in a tropical forest. Ecology Letters 18: 1397–1405. doi:10.1111/ele.12536. 26472095

[CIT0008] Barea JM , Pozo MJ, Azcón R, Azcón-Aguilar C. 2005. Microbial co-operation in the rhizosphere. Journal of Experimental Botany 56: 1761–1778. doi:10.1093/jxb/eri197. 15911555

[CIT0009] Bittebiere A-K , Vandenkoornhuyse P, Maluenda E, et al. 2020. Past spatial structure of plant communities determines arbuscular mycorrhizal fungal community assembly. Journal of Ecology 108: 546–560.

[CIT0010] Blanchet FG , Legendre P, Borcard D. 2008. Forward selection of explanatory variables. Ecology 89: 2623–2632. doi:10.1890/07-0986.1. 18831183

[CIT0011] Bokulich NA , Subramanian S, Faith JJ, et al. 2013. Quality-filtering vastly improves diversity estimates from Illumina amplicon sequencing. Nature Methods 10: 57–59. doi:10.1038/nmeth.2276. 23202435 PMC3531572

[CIT0012] Borcard D , Legendre P. 2002. All-scale spatial analysis of ecological data by means of principal coordinates of neighbour matrices. Ecological Modelling 153: 51–68. doi:10.1016/s0304-3800(01)00501-4.

[CIT0013] Breshears DD , Nyhan JW, Heil CE, Wilcox BP. 1998. Effects of woody plants on microclimate in a semiarid woodland: soil temperature and evaporation in canopy and intercanopy patches. International Journal of Plant Sciences 159: 1010–1017. doi:10.1086/314083.

[CIT0014] Brundrett MC. 2002. Coevolution of roots and mycorrhizas of land plants. New Phytologist 154: 275–304. doi:10.1046/j.1469-8137.2002.00397.x. 33873429

[CIT0015] Brundrett MC , Tedersoo L. 2018. Evolutionary history of mycorrhizal symbioses and global host plant diversity. New Phytologist 220: 1108–1115. doi:10.1111/nph.14976. 29355963

[CIT0016] Burnham KP , Anderson DR. 2002. Model selection and multimodel inference: a practical information-theoretic approach. New York: Springer.

[CIT0017] Cabal C , Deurwaerder HD, Matesanz S. 2021. Field methods to study the spatial root density distribution of individual plants. Plant and Soil 462: 25–43.

[CIT0018] Caporaso JG , Kuczynski J, Stombaugh J, et al. 2010. QIIME allows analysis of high-throughput community sequencing data. Nature Methods 7: 335–336. doi:10.1038/nmeth.f.303. 20383131 PMC3156573

[CIT0019] Chen L , Zheng Y, Gao C, et al. 2017. Phylogenetic relatedness explains highly interconnected and nested symbiotic networks of woody plants and arbuscular mycorrhizal fungi in a Chinese subtropical forest. Molecular Ecology 26: 2563–2575. doi:10.1111/mec.14061. 28207957

[CIT0020] Chesson P. 2000. Mechanisms of maintenance of species diversity. Annual Review of Ecology and Systematics 31: 343–366. doi:10.1146/annurev.ecolsys.31.1.343.

[CIT0021] Davison J , Moora M, Öpik M, et al. 2015. Global assessment of arbuscular mycorrhizal fungus diversity reveals very low endemism. Science 349: 970–973. doi:10.1126/science.aab1161. 26315436

[CIT0022] Davison J , Öpik M, Zobel M, Vasar M, Metsis M, Moora M. 2012. Communities of arbuscular mycorrhizal fungi detected in forest soil are spatially heterogeneous but do not vary throughout the growing season. PLoS One 7: e41938. doi:10.1371/journal.pone.0041938. 22879900 PMC3413688

[CIT0023] Davison J , Moora M, Semchenko M, et al. 2021. Temperature and pH define the realised niche space of arbuscular mycorrhizal fungi. New Phytologist 231: 763–776. 33507570 10.1111/nph.17240

[CIT0024] Debastiani VJ , Pillar VD. 2012. Syncsa-R tool for analysis of metacommunities based on functional traits and phylogeny of the community components. Bioinformatics 28: 2067–2068. doi:10.1093/bioinformatics/bts325. 22668789

[CIT0025] Edgar RC , Haas BJ, Clemente JC, Quince C, Knight R. 2011. UCHIME improves sensitivity and speed of chimera detection. Bioinformatics 27: 2194–2200. doi:10.1093/bioinformatics/btr381. 21700674 PMC3150044

[CIT0026] Esling P , Lejzerowicz F, Pawlowski J. 2015. Accurate multiplexing and filtering for high-throughput amplicon-sequencing. Nucleic Acids Research 43: 2513–2524. doi:10.1093/nar/gkv107. 25690897 PMC4357712

[CIT0027] García de León D , Moora M, Öpik M, et al. 2016. Symbiont dynamics during ecosystem succession: co-occurring plant and arbuscular mycorrhizal fungal communities. FEMS Microbiology Ecology 92: fiw097. doi:10.1093/femsec/fiw097. 27162183

[CIT0028] Hair JF , Black WC, Babin BJ, Anderson RE, Tatham RL. 2014. *Multivariate data analysis*. Harlow: Pearson Education.

[CIT0029] Harrell FE , Lee KL, Califf RM, Pryor DB, Rosati RA. 1984. Regression modelling strategies for improved prognostic prediction. Statistics in Medicine 3: 143–152. doi:10.1002/sim.4780030207. 6463451

[CIT0030] Hart MM , Reader RJ. 2002. Taxonomic basis for variation in the colonization strategy of arbuscular mycorrhizal fungi. New Phytologist 153: 335–344. doi:10.1046/j.0028-646x.2001.00312.x.

[CIT0031] Harvey PH , Pagel MD. 1991. The comparative method in evolutionary biology. Trends in Ecology & Evolution 239: 239.

[CIT0032] Hausmann NT , Hawkes CV. 2009. Plant neighborhood control of arbuscular mycorrhizal community composition. New Phytologist 183: 1188–1200. doi:10.1111/j.1469-8137.2009.02882.x. 19496954

[CIT0033] Helgason T , Merryweather JW, Young JPW, Fitter AH. 2007. Specificity and resilience in the arbuscular mycorrhizal fungi of a natural woodland community. Journal of Ecology 95: 623–630. doi:10.1111/j.1365-2745.2007.01239.x.

[CIT0034] Hiiesalu I , Öpik M, Metsis M, et al. 2012. Plant species richness belowground: higher richness and new patterns revealed by next-generation sequencing. Molecular Ecology 21: 2004–2016. doi:10.1111/j.1365-294X.2011.05390.x. 22168247

[CIT0035] Horn S , Hempel S, Verbruggen E, Rillig MC, Caruso T. 2017. Linking the community structure of arbuscular mycorrhizal fungi and plants: a story of interdependence? The ISME Journal 11: 1400–1411. doi:10.1038/ismej.2017.5. 28244977 PMC5437357

[CIT0036] Illuminati A , López-Angulo J, de la Cruz M, et al. 2021. Larger aboveground neighbourhood scales maximise similarity but do not eliminate discrepancies with belowground plant diversity in a Mediterranean shrubland. Plant and Soil 460: 497–509.

[CIT0037] Jamiołkowska A , Ksiȩzniak A, Gałązka A, Hetman B, Kopacki M, Skwaryło-Bednarz B. 2018. Impact of abiotic factors on development of the community of arbuscular mycorrhizal fungi in the soil: a review. International Agrophysics 32: 133–140.

[CIT0038] Javaid A. 2008. Allelopathy in mycorrhizal symbiosis in the Poaceae family. Allelopathy Journal 21: 207–218.

[CIT0039] Johnson D , Vandenkoornhuyse PJ, Leake JR, et al. 2004. Plant communities affect arbuscular mycorrhizal fungal diversity and community composition in grassland microcosms. New Phytologist 161: 503–515. 33873500 10.1046/j.1469-8137.2003.00938.x

[CIT0040] Jones FA , Erickson DL, Bernal MA, et al. 2011. The roots of diversity: below ground species richness and rooting distributions in a tropical forest revealed by DNA barcodes and inverse modeling. PLoS One 6: e24506. doi:10.1371/journal.pone.0024506. 21949723 PMC3176281

[CIT0041] Josse J , Husson F. 2012. Selecting the number of components in principal component analysis using cross-validation approximations. Computational Statistics and Data Analysis 56: 1869–1879. doi:10.1016/j.csda.2011.11.012.

[CIT0042] Khalvati MA , Hu Y, Mozafar A, Schmidhalter U. 2005. Quantification of water uptake by arbuscular mycorrhizal hyphae and its significance for leaf growth, water relations, and gas exchange of barley subjected to drought stress. Plant Biology 7: 706–712. doi:10.1055/s-2005-872893. 16388474

[CIT0043] Kiers ET , Duhamel M, Beesetty Y, et al. 2011. Reciprocal rewards stabilize cooperation in the mycorrhizal symbiosis. Science 333: 880–882. doi:10.1126/science.1208473. 21836016

[CIT0044] Kivlin SN , Hawkes CV, Treseder KK. 2011. Global diversity and distribution of arbuscular mycorrhizal fungi. Soil Biology and Biochemistry 43: 2294–2303. doi:10.1016/j.soilbio.2011.07.012.

[CIT0045] Klimešová J , Klimeš L. 2007. Bud banks and their role in vegetative regeneration – a literature review and proposal for simple classification and assessment. Perspectives in Plant Ecology, Evolution and Systematics 8: 115–129.

[CIT0046] Knegt B , Jansa J, Franken O, et al. 2016. Host plant quality mediates competition between arbuscular mycorrhizal fungi. Fungal Ecology 20: 233–240. doi:10.1016/j.funeco.2014.09.011.

[CIT0047] Koch AM , Antunes PM, Maherali H, Hart MM, Klironomos JN. 2017. Evolutionary asymmetry in the arbuscular mycorrhizal symbiosis: conservatism in fungal morphology does not predict host plant growth. New Phytologist 214: 1330–1337. doi:10.1111/nph.14465. 28186629

[CIT0048] Koorem K , Tulva I, Davison J, et al. 2017. Arbuscular mycorrhizal fungal communities in forest plant roots are simultaneously shaped by host characteristics and canopy-mediated light availability. Plant and Soil 410: 259–271.

[CIT0049] Kress WJ , Erickson DL, Jones FA, et al. 2009. Plant DNA barcodes and a community phylogeny of a tropical forest dynamics plot in Panama. Proceedings of the National Academy of Sciences of the United States of America 106: 18621–18626. doi:10.1073/pnas.0909820106. 19841276 PMC2763884

[CIT0050] Lambers H , Shane MW, Cramer MD, Pearse SJ, Veneklaas EJ. 2006. Root structure and functioning for efficient acquisition of phosphorus: matching morphological and physiological traits. Annals of Botany 98: 693–713. doi:10.1093/aob/mcl114. 16769731 PMC2806175

[CIT0051] Lee J , Lee S, Young JPW. 2008. Improved PCR primers for the detection and identification of arbuscular mycorrhizal fungi. FEMS Microbiology Ecology 65: 339–349. doi:10.1111/j.1574-6941.2008.00531.x. 18631176

[CIT0052] Legay N , Baxendale C, Grigulis K, et al. 2014. Contribution of above- and below-ground plant traits to the structure and function of grassland soil microbial communities. Annals of Botany 114: 1011–1021. doi:10.1093/aob/mcu169. 25122656 PMC4171078

[CIT0053] Legendre P , Gallagher ED. 2001. Ecologically meaningful transformations for ordination of species data. Oecologia 129: 271–280. doi:10.1007/s004420100716. 28547606

[CIT0054] Legendre P , Legendre L. 2012. Numerical ecology. Amsterdam: Elsevier.

[CIT0055] Legendre P , Borcard D, Roberts DW. 2012. Variation partitioning involving orthogonal spatial eigenfunction submodels. Ecology 93: 1234–1240. doi:10.1890/11-2028.1. 22764509

[CIT0056] Levin RA , Wagner WL, Hoch PC, et al. 2003. Family-level relationships of Onagraceae based on chloroplast *rbcL* and *ndhF* data. American Journal of Botany 90: 107–115. doi:10.3732/ajb.90.1.107. 21659085

[CIT0057] Li X , Qi Z, Yu X, et al. 2021. Soil pH drives the phylogenetic clustering of the arbuscular mycorrhizal fungal community across subtropical and tropical pepper fields of China. Applied Soil Ecology 165: 103978. doi:10.1016/j.apsoil.2021.103978.

[CIT0058] Liu Y , Johnson NC, Mao L, et al. 2015. Phylogenetic structure of arbuscular mycorrhizal community shifts in response to increasing soil fertility. Soil Biology and Biochemistry 89: 196–205. doi:10.1016/j.soilbio.2015.07.007.

[CIT0059] López-Angulo J , de la Cruz M, Chacón-Labella J, et al. 2020. The role of root community attributes in predicting soil fungal and bacterial community patterns. New Phytologist 228: 1070–1082. 32557640 10.1111/nph.16754

[CIT0060] López-García A , Palenzuela J, Barea JM, Azcón-Aguilar C. 2014. Life-history strategies of arbuscular mycorrhizal fungi determine succession into roots of *Rosmarinus officinalis* L., a characteristic woody perennial plant species from Mediterranean ecosystems. Plant and Soil 379: 247–260. doi:10.1007/s11104-014-2060-6.

[CIT0061] López-García A , Varela-Cervero S, Vasar M, Öpik M, Barea JM, Azcón-Aguilar C. 2017. Plant traits determine the phylogenetic structure of arbuscular mycorrhizal fungal communities. Molecular Ecology 26: 6948–6959. doi:10.1111/mec.14403. 29110362

[CIT0062] Losos JB. 2008. Phylogenetic niche conservatism, phylogenetic signal and the relationship between phylogenetic relatedness and ecological similarity among species. Ecology Letters 11: 995–1003. doi:10.1111/j.1461-0248.2008.01229.x. 18673385

[CIT0063] Maestre FT , Cortina J. 2005. Remnant shrubs in Mediterranean semi-arid steppes: effects of shrub size, abiotic factors and species identity on understorey richness and occurrence. Acta Oecologica 27: 161–169. doi:10.1016/j.actao.2004.11.003.

[CIT0064] Magoč T , Salzberg SL. 2011. FLASH: fast length adjustment of short reads to improve genome assemblies. Bioinformatics 27: 2957–2963. doi:10.1093/bioinformatics/btr507. 21903629 PMC3198573

[CIT0065] Maherali H , Klironomos JN. 2007. Influence of phylogeny on fungal community assembly and ecosystem functioning. Science 316: 1746–1748. doi:10.1126/science.1143082. 17588930

[CIT0066] Matesanz S , Pescador DS, Pías B, et al. 2019. Estimating belowground plant abundance with DNA metabarcoding. Molecular Ecology Resources 19: 1265–1277. doi:10.1111/1755-0998.13049. 31232514

[CIT0067] Mayfield MM , Levine JM. 2010. Opposing effects of competitive exclusion on the phylogenetic structure of communities. Ecology Letters 13: 1085–1093. doi:10.1111/j.1461-0248.2010.01509.x. 20576030

[CIT0068] Mokany K , Raison RJ, Prokushkin AS. 2006. Critical analysis of root:shoot ratios in terrestrial biomes. Global Change Biology 12: 84–96.

[CIT0069] Montesinos-Navarro A , Segarra-Moragues JG, Valiente-Banuet A, Verdú M. 2015. Evidence for phylogenetic correlation of plant–AMF assemblages? Annals of Botany 115: 171–177. doi:10.1093/aob/mcu228. 25452252 PMC4551090

[CIT0070] Morgan BST , Egerton-Warburton LM. 2017. Barcoded NS31/AML2 primers for sequencing of arbuscular mycorrhizal communities in environmental samples. Applications in Plant Sciences 5: 1700017. 10.3732/apps.1700017 PMC5584815 28924511

[CIT0071] Neuenkamp L , Moora M, Öpik M, et al. 2018. The role of plant mycorrhizal type and status in modulating the relationship between plant and arbuscular mycorrhizal fungal communities. New Phytologist 220: 1236–1247. doi:10.1111/nph.14995. 29369351

[CIT0072] Neuenkamp L , Zobel M, Koorem K, et al. 2021. Light availability and light demand of plants shape the arbuscular mycorrhizal fungal communities in their roots. Ecology Letters 24: 426–437. doi:10.1111/ele.13656. 33319429

[CIT0073] Öpik M , Metsis M, Daniell TJ, Zobel M, Moora M. 2009. Large-scale parallel 454 sequencing reveals host ecological group specificity of arbuscular mycorrhizal fungi in a boreonemoral forest. New Phytologist 184: 424–437. doi:10.1111/j.1469-8137.2009.02920.x. 19558424

[CIT0074] Öpik M , Vanatoa A, Vanatoa E, et al. 2010. The online database Maarj*AM* reveals global and ecosystemic distribution patterns in arbuscular mycorrhizal fungi (Glomeromycota). New Phytologist 188: 223–241. doi:10.1111/j.1469-8137.2010.03334.x. 20561207

[CIT0075] Peek MS , Leffler AJ, Ivans CY, Ryel RJ, Caldwell MM. 2005. Fine root distribution and persistence under field conditions of three co-occurring Great Basin species of different life form. New Phytologist 165: 171–180. 15720631 10.1111/j.1469-8137.2004.01186.x

[CIT0076] Pillar VD , Duarte LdS. 2010. A framework for metacommunity analysis of phylogenetic structure. Ecology Letters 13: 587–596. doi:10.1111/j.1461-0248.2010.01456.x. 20337699

[CIT0077] Powell JR , Parrent JL, Hart MM, Klironomos JN, Rillig MC, Maherali H. 2009. Phylogenetic trait conservatism and the evolution of functional trade-offs in arbuscular mycorrhizal fungi. Proceedings of the Royal Society B: Biological Sciences 276: 4237–4245. doi:10.1098/rspb.2009.1015. PMC2821337 19740877

[CIT0078] Rao CR. 1982. Diversity and dissimilarity coefficients: a unified approach. Theoretical Population Biology 21: 24–43. doi:10.1016/0040-5809(82)90004-1.

[CIT0079] Reintal M , Tali K, Haldna M, Kull T. 2010. Habitat preferences as related to the prolonged dormancy of perennial herbs and ferns. Plant Ecology 210: 111–123. doi:10.1007/s11258-010-9742-9.

[CIT0080] Rognes T , Flouri T, Nichols B, Quince C, Mahé F. 2016. VSEARCH: a versatile open source tool for metagenomics. PeerJ 4: e2584. doi:10.7717/peerj.2584. 27781170 PMC5075697

[CIT0081] Sánchez-Blanco MJ , Ferrández T, Morales MA, Morte A, Alarcón JJ. 2004. Variations in water status, gas exchange, and growth in *Rosmarinus officinalis* plants infected with *Glomus deserticola* under drought conditions. Journal of Plant Physiology 161: 675–682. doi:10.1078/0176-1617-01191. 15266714

[CIT0082] Schenk HJ , Jackson RB. 2002. Rooting depths, lateral root spreads and below-ground/above-ground allometries of plants in water-limited ecosystems. Journal of Ecology 90: 480–494. doi:10.1046/j.1365-2745.2002.00682.x.

[CIT0083] Schliep KP. 2011. phangorn: phylogenetic analysis in R. Bioinformatics 27: 592–593. doi:10.1093/bioinformatics/btq706. 21169378 PMC3035803

[CIT0084] Simon L , Lalonde M, Bruns TD. 1992. Specific amplification of 18S fungal ribosomal genes from vesicular-arbuscular endomycorrhizal fungi colonizing roots. Applied and Environmental Microbiology 58: 291–295. doi:10.1128/aem.58.1.291-295.1992. 1339260 PMC195206

[CIT0085] Šmilauer P , Košnar J, Kotilínek M, Šmilauerová M. 2020. Contrasting effects of host identity, plant community, and local species pool on the composition and colonization levels of arbuscular mycorrhizal fungal community in a temperate grassland. New Phytologist 225: 461–473. 31408907 10.1111/nph.16112

[CIT0086] Smith SE , Read DJ. 2010. Mycorrhizal symbiosis. Cambridge, UK: Academic Press.

[CIT0087] Soil Survey Staff. 2014. Keys to soil taxonomy. Blacksburg: Pocahontas Press.

[CIT0088] Spatafora JW , Chang Y, Benny GL, et al. 2016. A phylum-level phylogenetic classification of zygomycete fungi based on genome-scale data. Mycologia 108: 1028–1046. doi:10.3852/16-042. 27738200 PMC6078412

[CIT0089] Sun K , McCormack ML, Li L, Ma Z, Guo D. 2016. Fast-cycling unit of root turnover in perennial herbaceous plants in a cold temperate ecosystem. Scientific Reports 6: 19698. doi:10.1038/srep19698. 26791578 PMC4726329

[CIT0090] Teste FP , Jones MD, Dickie IA. 2020. Dual-mycorrhizal plants: their ecology and relevance. New Phytologist 225: 1835–1851. 31514244 10.1111/nph.16190

[CIT0091] Thonar C , Frossard E, Šmilauer P, Jansa J. 2014. Competition and facilitation in synthetic communities of arbuscular mycorrhizal fungi. Molecular Ecology 23: 733–746. doi:10.1111/mec.12625. 24330316

[CIT0092] Torrecillas E , Alguacil MM, Roldán A. 2012. Host preferences of arbuscular mycorrhizal fungi colonizing annual herbaceous plant species in semiarid mediterranean prairies. Applied and Environmental Microbiology 78: 6180–6186. doi:10.1128/AEM.01287-12. 22752164 PMC3416610

[CIT0093] Träger S , Öpik M, Vasar M, Wilson SD. 2019. Belowground plant parts are crucial for comprehensively estimating total plant richness in herbaceous and woody habitats. Ecology 100: e02575. doi:10.1002/ecy.2575. 30516275

[CIT0094] Trinder CJ , Johnson D, Artz RRE. 2009. Litter type, but not plant cover, regulates initial litter decomposition and fungal community structure in a recolonising cutover peatland. Soil Biology and Biochemistry 41: 651–655.

[CIT0095] Valverde-Barrantes OJ , Horning AL, Smemo KA, Blackwood CB. 2016. Phylogenetically structured traits in root systems influence arbuscular mycorrhizal colonization in woody angiosperms. Plant and Soil 404: 1–12. doi:10.1007/s11104-016-2820-6.

[CIT0096] Vályi K , Mardhiah U, Rillig MC, Hempel S. 2016. Community assembly and coexistence in communities of arbuscular mycorrhizal fungi. The ISME Journal 10: 2341–2351. doi:10.1038/ismej.2016.46. 27093046 PMC5030697

[CIT0097] Vandenkoornhuyse P , Husband R, Daniell TJ, et al. 2002. Arbuscular mycorrhizal community composition associated with two plant species in a grassland ecosystem. Molecular Ecology 11: 1555–1564. doi:10.1046/j.1365-294x.2002.01538.x. 12144674

[CIT0098] Van Der Heijden MGA , Horton TR. 2009. Socialism in soil? The importance of mycorrhizal fungal networks for facilitation in natural ecosystems. Journal of Ecology 97: 1139–1150. doi:10.1111/j.1365-2745.2009.01570.x.

[CIT0099] Van Der Heijden MGA , Klironomos JN, Ursic M, et al. 1998. Mycorrhizal fungal diversity determines plant biodiversity, ecosystem variability and productivity. Nature 396: 69–72. doi:10.1038/23932.

[CIT0100] van der Heijden MGA , Martin FM, Selosse MA, Sanders IR. 2015. Mycorrhizal ecology and evolution: the past, the present, and the future. New Phytologist 205: 1406–1423. 25639293 10.1111/nph.13288

[CIT0101] Van Geel M , Jacquemyn H, Plue J, et al. 2018. Abiotic rather than biotic filtering shapes the arbuscular mycorrhizal fungal communities of European seminatural grasslands. New Phytologist 220: 1262–1272. 29243832 10.1111/nph.14947

[CIT0102] Weigelt A , Mommer L, Andraczek K, et al. 2021. An integrated framework of plant form and function: the belowground perspective. New Phytologist 232: 42–59. doi:10.1111/nph.17590. 34197626

[CIT0103] Wilson GWT , Rice CW, Rillig MC, Springer A, Hartnett DC. 2009. Soil aggregation and carbon sequestration are tightly correlated with the abundance of arbuscular mycorrhizal fungi: results from long-term field experiments. Ecology Letters 12: 452–461. doi:10.1111/j.1461-0248.2009.01303.x. 19320689

[CIT0104] Xia M , Valverde-Barrantes OJ, Suseela V, Blackwood CB, Tharayil N. 2021. Coordination between compound-specific chemistry and morphology in plant roots aligns with ancestral mycorrhizal association in woody angiosperms. New Phytologist 232: 1259–1271. 34137048 10.1111/nph.17561

[CIT0105] Xu T , Veresoglou SD, Chen Y, et al. 2016. Plant community, geographic distance and abiotic factors play different roles in predicting AMF biogeography at the regional scale in northern China. Environmental Microbiology Reports 8: 1048–1057. doi:10.1111/1758-2229.12485. 27718332

[CIT0106] Yang H , Zang Y, Yuan Y, Tang J, Chen X. 2012. Selectivity by host plants affects the distribution of arbuscular mycorrhizal fungi: evidence from ITS rDNA sequence metadata. BMC Evolutionary Biology 12: 50. doi:10.1168/1471-2148-12-50. 22498355 PMC3395829

[CIT0107] Yang H , Dai Y, Wang X, Zhang Q, Zhu L, Bian X. 2014. Meta-analysis of interactions between arbuscular mycorrhizal fungi and biotic stressors of plants. The Scientific World Journal 2014: 746506. doi:10.1155/2014/746506. 24558327 PMC3914602

[CIT0108] Zhu Y-G , Miller RM. 2003. Carbon cycling by arbuscular mycorrhizal fungi in soil–plant systems. Trends in Plant Science 8: 407–409. doi:10.1016/s1360-1385(03)00184-5. 13678905

[CIT0109] Zuur AF , Ieno EN, Elphick CS. 2010. A protocol for data exploration to avoid common statistical problems. Methods in Ecology and Evolution 1: 3–14.

