# Supplementary material for: Ecological drivers of fine-scale distribution of arbuscular mycorrhizal fungi in a semiarid Mediterranean scrubland
Source: Ann Bot. 2023 Mar 28;131(7):1107–19. doi: 10.1093/aob/mcad050 (PMC10457037; doi:10.1093/aob/mcad050)
Supplement: mcad050_suppl_Supplementary_Material [file mcad050_suppl_supplementary_material.docx]

## **Supporting Information**

**Ecological drivers of fine-scale distribution of arbuscular mycorrhizal fungi in a semiarid Mediterranean scrubland**

Authors: Jesús López-Angulo, Silvia Matesanz, Angela Illuminati, David S. Pescador, Ana M. Sánchez, Beatriz Pías, Julia Chacón-Labella, Marcelino de la Cruz, and Adrián Escudero

**Methods S1** R packages used for specific applications.

We used the function ‘matrix.p’ of the ‘SYNCSA’ R package (Debastiani & Pillar, 2012) to compute the phylogenetic composition of arbuscular mycorrhizal (AM) fungal and plant communities as the AM fungal phylogenetically-weighted species composition matrix (Pillar & Duarte, 2010) by weighting the AM fungal abundances by their phylogenetic relationships estimated from the phylogenetic trees. Rao was computed using the function ‘melodic’ (de Bello *et al.*, 2016) from the matrix of pairwise phylogenetic distances (function ‘cophenetic’ of the ‘stats’ package). The function ’melodic’ computes Rao as:

$$Rao= \sum_{i=1}^{SR} \sum_{j=1}^{SR} d_{ij}p_{i}p_{j}$$

where *d_ij_* is the phylogenetic distance between plant species *i* and *j*, and *p_i_* and *p_j_* are the number of OTU of taxa *i* and *j*, respectively, after square root transformation.

We used several functions from the ‘vegan’ R package (Oksanen *et al.*, 2019): we used the function ‘rarecurve’ to estimate the rarefaction curves; the functions ‘vegdist’ and ‘metaMDS’ to compute the dissimilarity matrices and the non-metric multidimensional scaling (nMDS) ordinations, respectively; the function ‘rda’ to computed the partial redundancy analyses. To select the number of components in PCA, we used the smoothing approximation of the cross-validation criterion with the function ‘estim_ncp’ from the FactoMineR R package (Josse & Husson, 2012). We used the functions ‘dbmem’ and ‘forward.sel’ from the ‘adespatial’ R package (Dray *et al.*, 2016) to compute the distance-based Moran’s eigenvectors (dbMEM; Legendre & Legendre, 2012) and the forward selection, respectively. We used the function ‘vif’ of the ‘car’ package (Fox & Weisberg, 2011) to assess the absence of multicollinearity in all models using the variance inflation factor, setting a limit of VIF ≤ 4 (Zuur *et al.*, 2010). We used the ‘glm’ function of the ‘stats’ R package to fit the Generalized linear models. We used the functions ‘dredge’ from the R package ‘MuMIn (Bartoń, 2013) to perform Model selection procedure and the argument ‘m.lim‘ to avoid overparameterization and to allow a maximum of seven predictors in the candidate models. We used the ‘mantel’ function in ‘ecodist’ R package (Goslee & Urban, 2007) to perform the partial Mantel tests.

**References**

Bartoń K. 2013. MuMIn: Multi-Model Inference.

de Bello F, Carmona CP, Lepš J, Szava-Kovats R, Pärtel M. 2016. Functional diversity through the mean trait dissimilarity: resolving shortcomings with existing paradigms and algorithms. *Oecologia* 180: 933–940.

Debastiani VJ, Pillar VD. 2012. Syncsa-R tool for analysis of metacommunities based on functional traits and phylogeny of the community components. *Bioinformatics* 28: 2067–2068.

Dray S, Blanchet G, Borcard D, Guenard G, Jombart T, Larocque G, Legendre P, Madi N, Wagner HH. 2016. Adespatial: multivariate multiscale spatial analysis. R package version.

Fox J, Weisberg S. 2011. An R companion to applied regression. *Vienna: R Foundation for Statistical Computing*.

Goslee SC, Urban DL. 2007. The ecodist package for dissimilarity-based analysis of ecological data. *Journal of Statistical Software* 22: 1–19.

Josse, J., & Husson, F. 2012. Selecting the number of components in principal component analysis using cross-validation approximations. Computational Statistics and Data Analysis, 56(6), 1869–1879.

Legendre P, Legendre L. 2012. *Numerical ecology*. Amsterdam, the Netherlands: Elsevier.

Lê S., Josse J, Husson F. 2008. FactoMineR: A Package for Multivariate Analysis. *Journal of Statistical Software*, 25(1), 1–18. doi:10.18637/jss.v025.i01.

Oksanen J, Blanchet FG, Friendly M, Kindt R, Legendre P, McGlinn D, Minchin PR, O’Hara RB, Simpson GL, Solymos P, *et al.* 2019. *Vegan: community ecology package. R package version 2.5-6*.

Pillar VD, Duarte L d. S. 2010. A framework for metacommunity analysis of phylogenetic structure. *Ecology Letters* 13: 587–596.

Zuur AF, Ieno EN, Elphick CS. 2010. A protocol for data exploration to avoid common statistical problems. *Methods in Ecology and Evolution* 1: 3–14.

**Fig. S1** Core sampling design. Sampling consisted of 64 sampling units (blue circles) laid out on an 8 × 8 m regular grid. Twenty additional sampling units (red triangles) were established to consider a finer spatial scale.


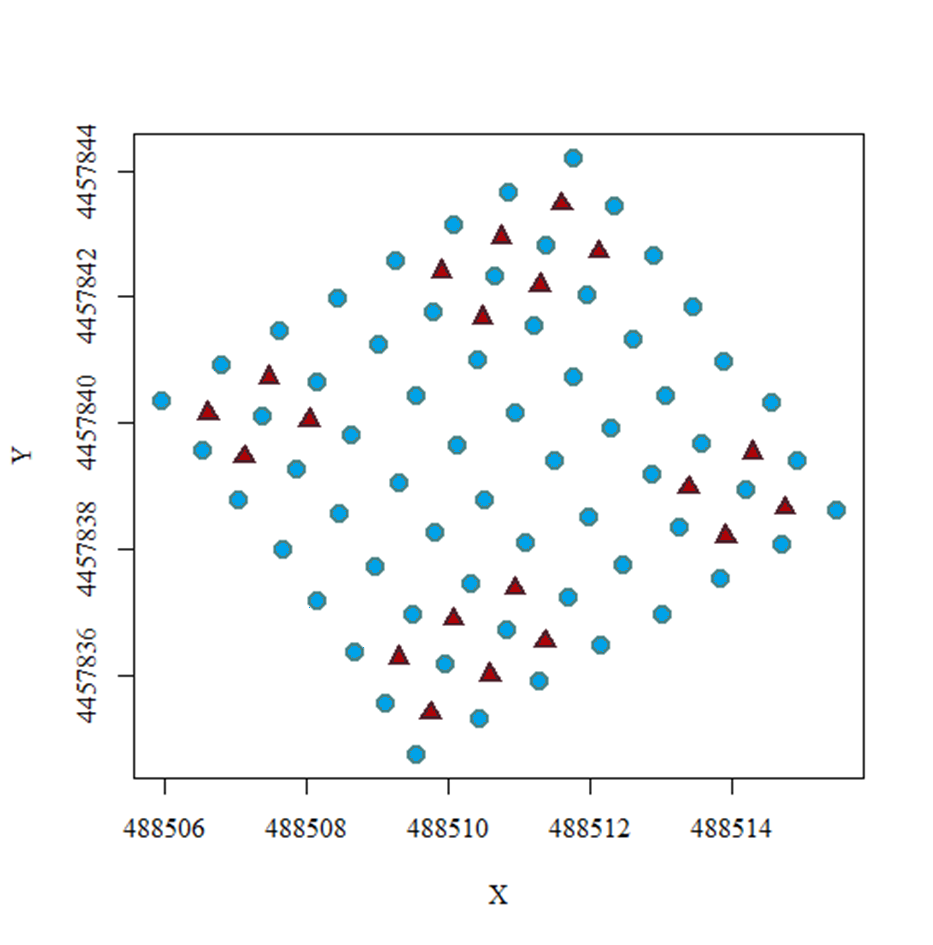


**Fig. S2** Rarefaction curve of arbuscular mycorrhizal fungal (AMF) communities for each soil sample.


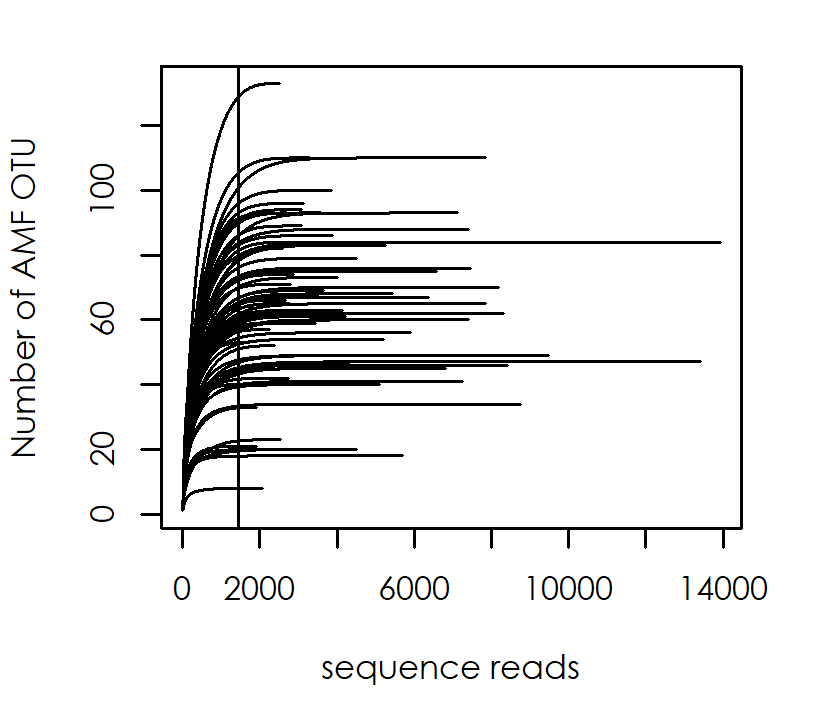


**Fig. S3** Non-metric multidimensional scaling (nMDS) ordinations showing patterns of variation in plant aboveground taxonomic (a), aboveground phylogenetic (b), belowground taxonomic (c), and belowground phylogenetic (d) composition based on 73 sample units (samples from the sampling circles for the aboveground plant community and soil samples for the belowground plant community). Grey points indicate the position of the samples in the multidimensional space. See Table S1 in Supplementary material for abbreviations of species names.


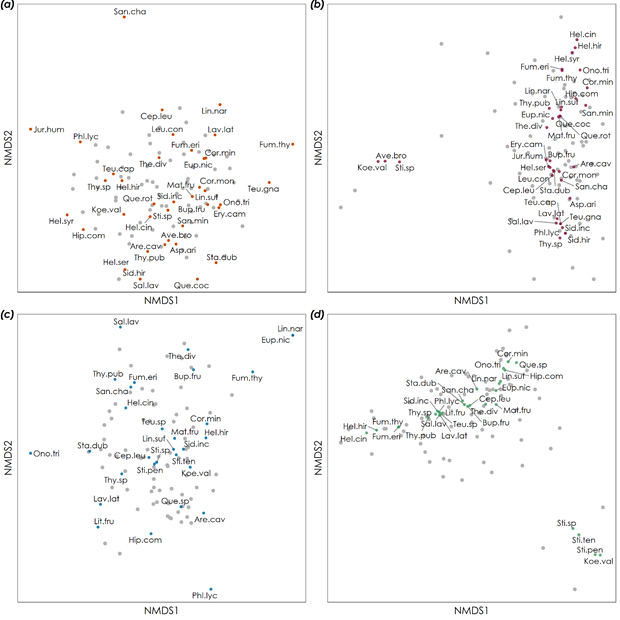


**Fig. S4** Pairwise scatterplots of the plant community taxonomic attributes. The panels on the diagonal represent the distribution of each attribute. The panels below the diagonal show the relationship between pairs of taxonomic attributes, and the panels above the diagonal show the Pearson correlation coefficient. Abbreviations: BTC, belowground taxonomic composition; ATC, aboveground taxonomic composition; BTD, belowground taxonomic diversity; ATD, aboveground taxonomic diversity.

**
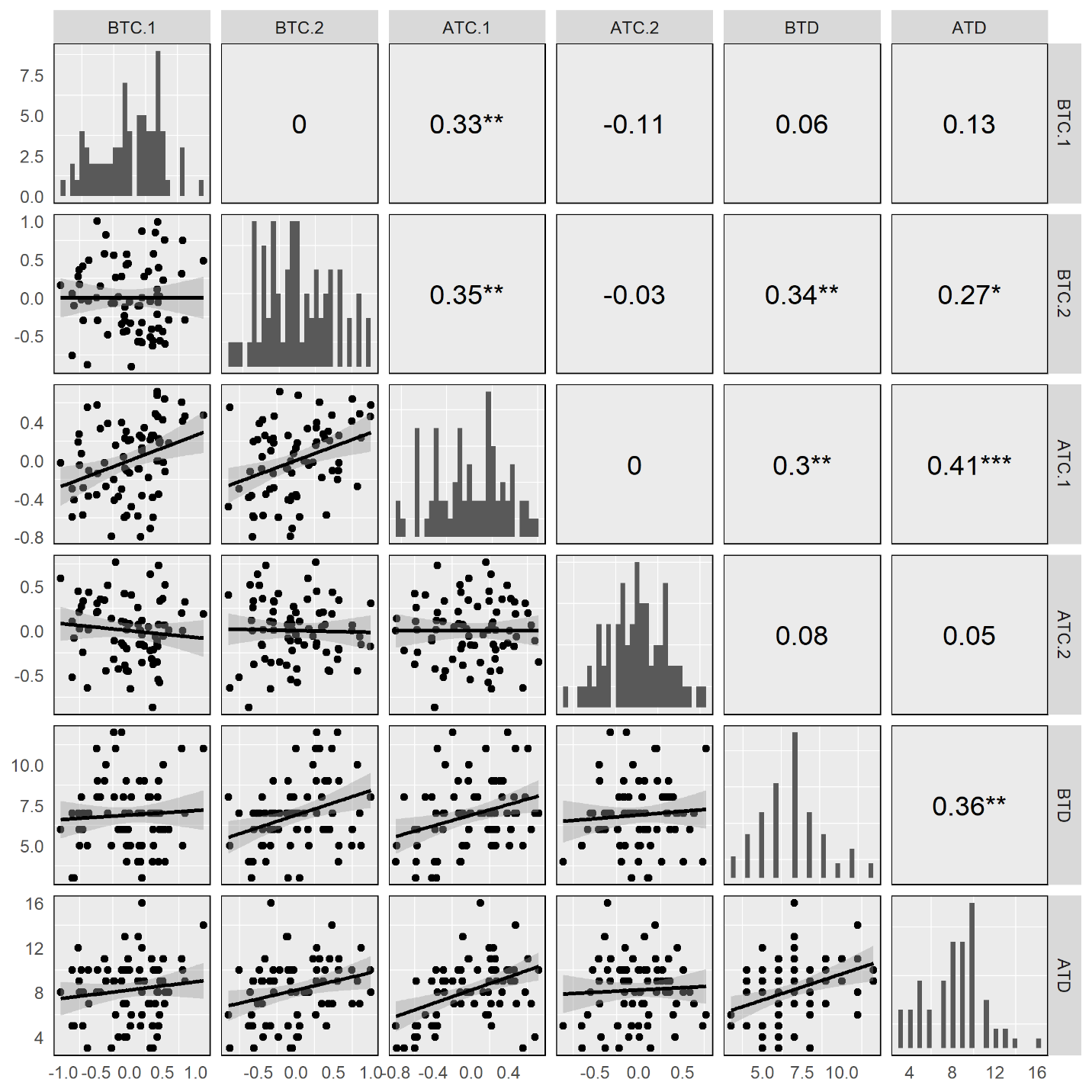
Fig. S5** Pairwise scatterplots of the plant community phylogenetic attributes. The panels on the diagonal represent the distribution of each attribute. The panels below the diagonal show the relationship between pairs of phylogenetic attributes, and the panels above the diagonal show the Pearson correlation coefficient. Abbreviations: BPC, belowground phylogenetic composition; APC, aboveground phylogenetic composition; BPD, belowground phylogenetic diversity; APD, aboveground phylogenetic diversity.

**
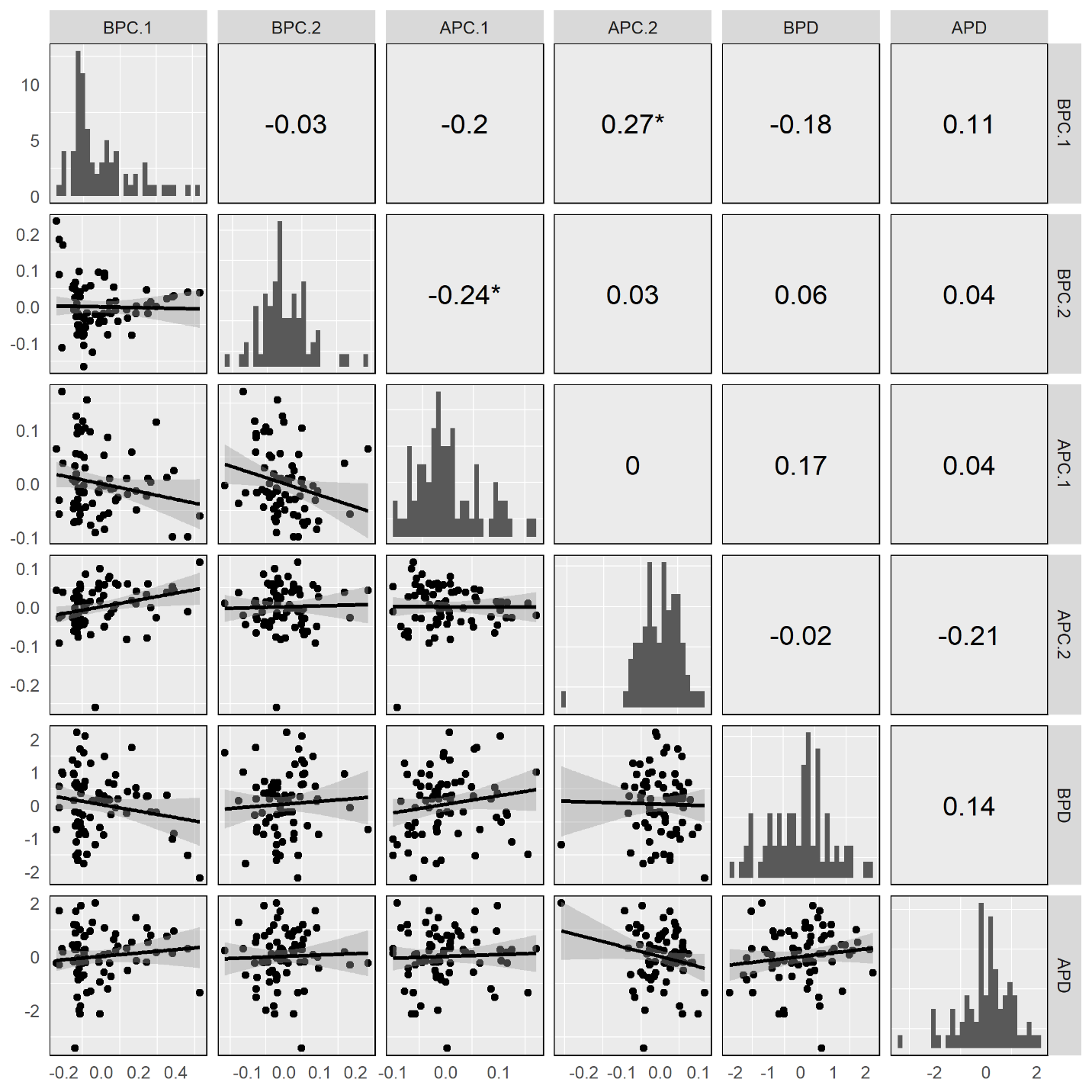
**

**Fig. S6** Proportion of sequence reads of arbuscular mycorrhizal fungi (AMF) at the family level across all samples (n = 73).


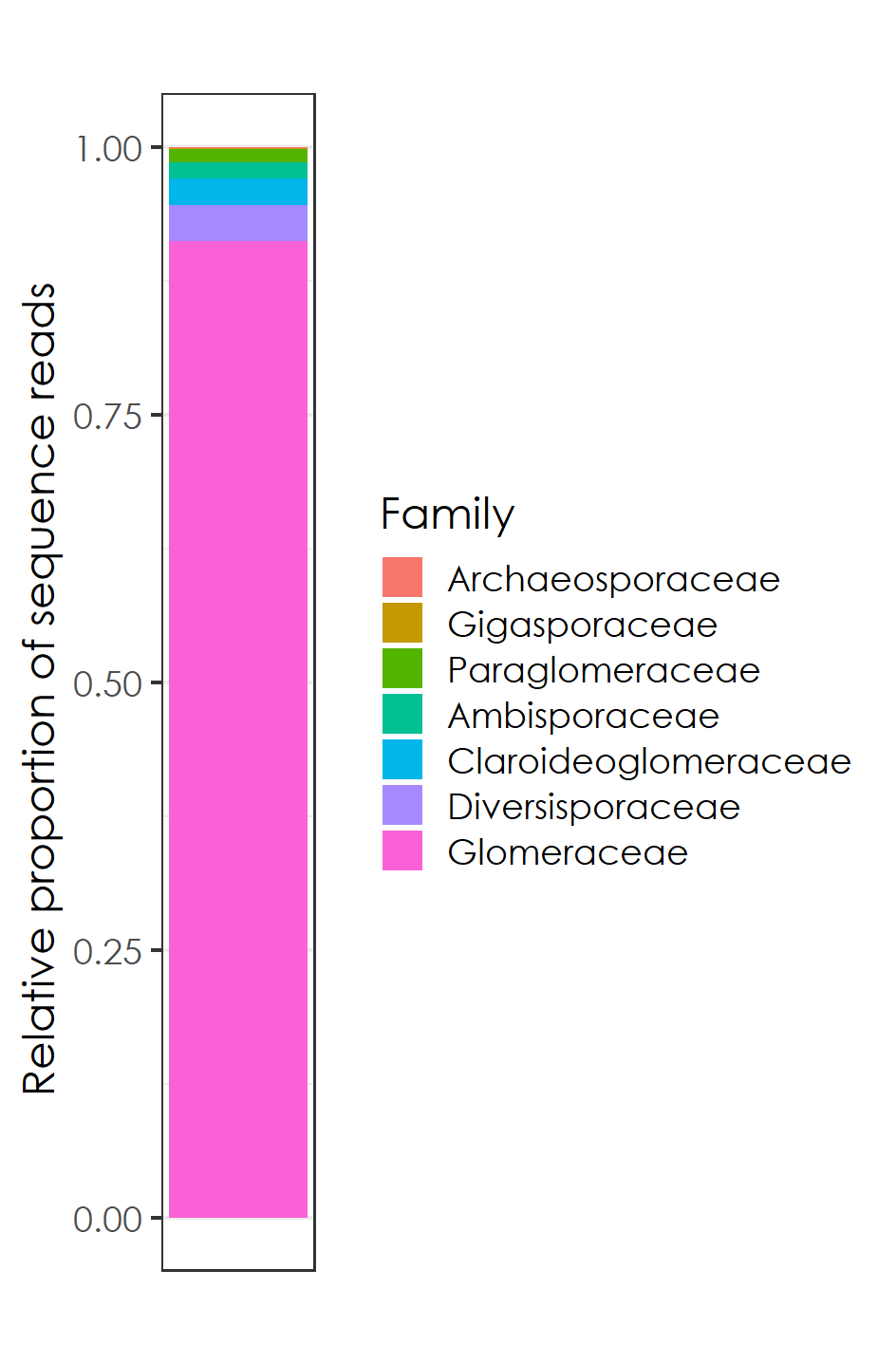


**Fig. S7** Venn diagrams showing variance partitioning results of AM fungal taxonomic (a) and phylogenetic (b) composition (number of OTUs), and taxonomic (c) and phylogenetic (d) diversity explained by plant community attributes (plant), soil properties (soil) and spatial covariates (space). The reported values are adjusted R2, representing the unique and shared variance explained by each predictor. Areas and intersections without values represent 0% explained and 0% shared variance, respectively.


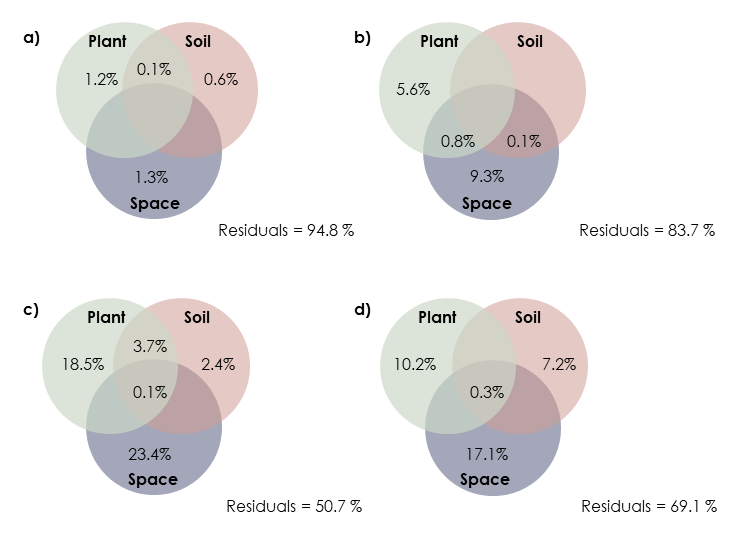


**Fig. S8** Venn diagrams showing variance partitioning results of arbuscular mycorrhizal (AM) fungal taxonomic (a) and phylogenetic (b) composition, and taxonomic (c) and phylogenetic (d) diversity explained by the belowground (below) and aboveground (above) taxonomic and phylogenetic attributes of the plant community. The variables of each set of predictors that were included in the variance partitioning analysis for AM fungal composition were selected applying a forward selection with double-stopping criterion, while for AM fungal diversity, they were selected using a model selection procedure based on the sum of Akaike weights (see Supplementary Data Table S5 for the model selection). The reported values are adjusted R2, representing the unique and shared variance explained by each predictor. Areas and intersections without values represent 0% explained and 0% shared variance, respectively.


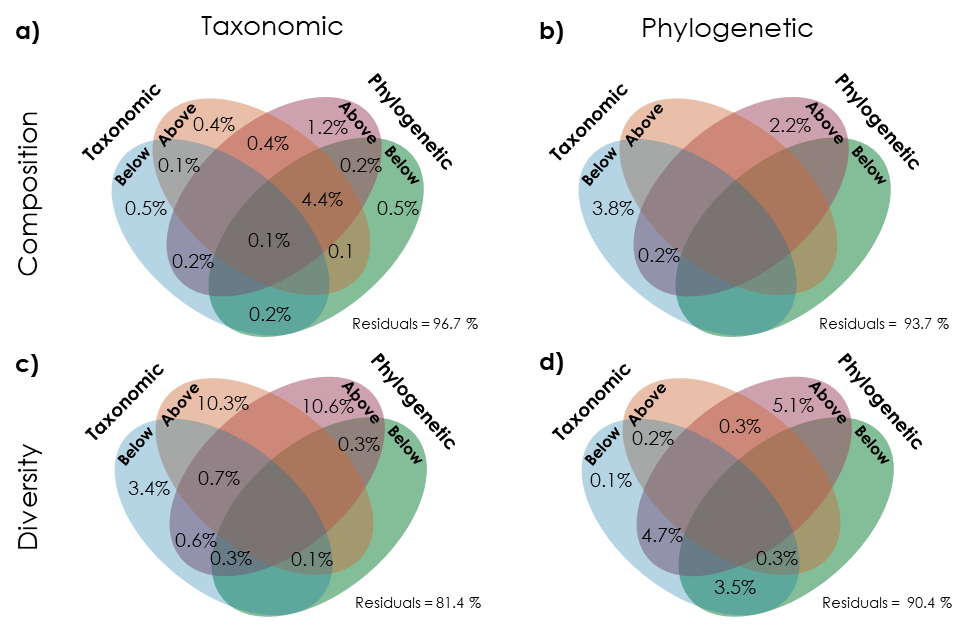


**Fig. S9** Redundancy analysis (RDA) biplot showing the relationship of AM fungal taxonomic composition (a) and phylogenetic composition (b) with the taxonomic and phylogenetic plant community attributes. Only the significant plant community attributes (P < 0.05) are shown (see Table 1). Points represent AM fungal OTUs and different colours represent different AM fungal families. Glomeraceae points are semi-transparent to make overlapping points visible. Note the small black arrow corresponding to aboveground taxonomic diversity almost overlapping belowground taxonomic composition.1. Composition.1 and composition.2 are the scores of each sample on the two first axes of nonmetric multidimensional scaling ordinations (nMDS; Fig. S3). Abbreviations: Belowgr., belowground; Abovegr., aboveground.

**
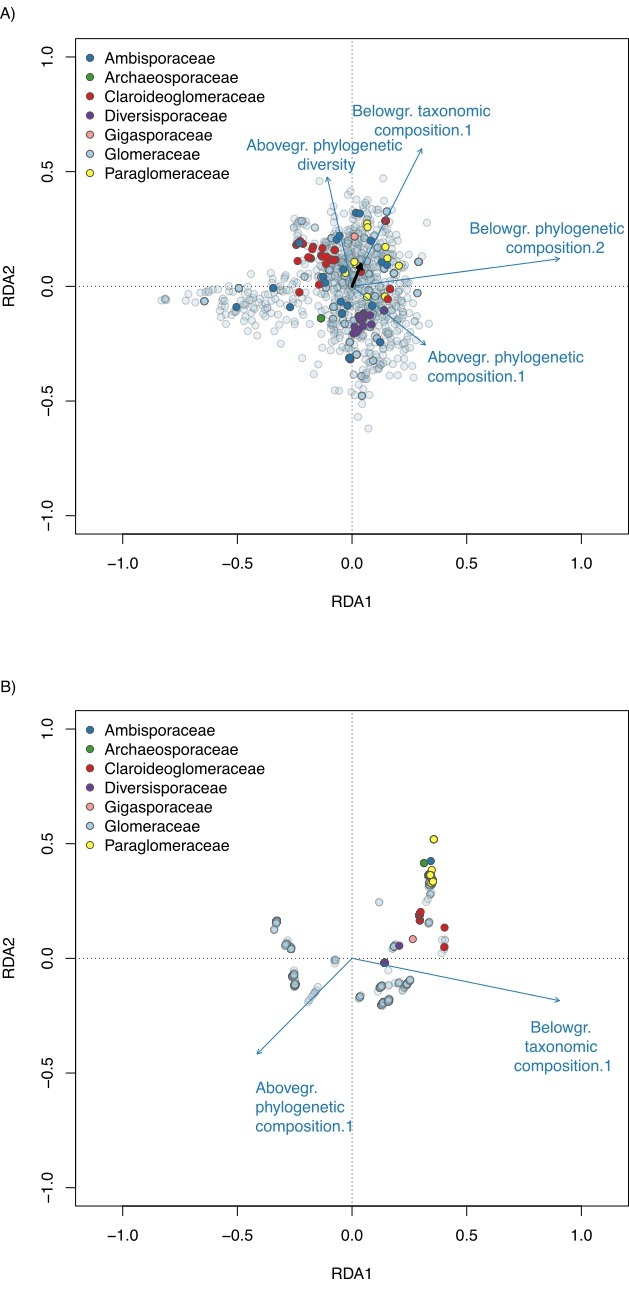
**

**Fig. S10** Redundancy analysis ordination (RDA) plot showing the relationship of AM fungal (a) taxonomic composition and (b) phylogenetic composition to soil physicochemical properties and the spatial covariates. Only the variables that significantly explained variability in AM fungi composition (P < 0.05) are shown (Table S3). Different colours represent different AM fungal families. Glomeraceae points are semi-transparent to make overlapping points visible. Note that composition.1 and composition.2 are variables calculated as the scores of each sample on the two first axes of nonmetric multidimensional scaling ordinations (nMDS; Fig. S3). Abbreviations: dbMEM, distance-based Moran’s eigenvectors maps.

**
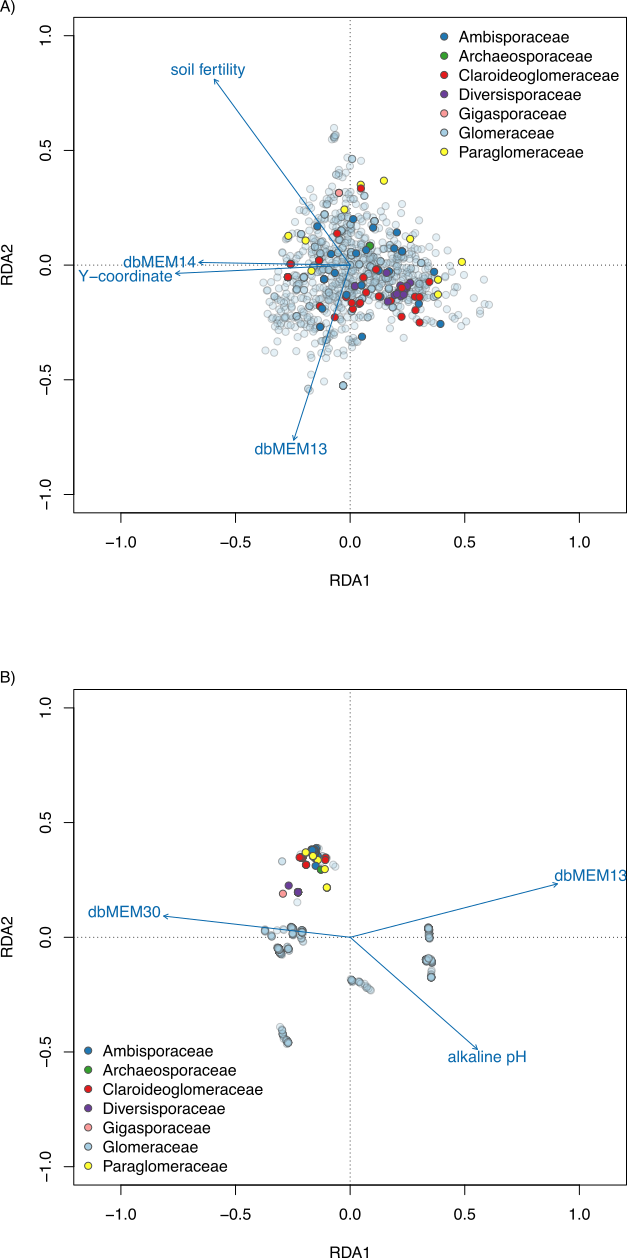
**

**Fig**. **S11** Linear relationships between the AM fungal taxonomic diversity (number of OTUs after square root transformation) of each AM fungal family and the taxonomic and phylogenetic plant community attributes.

**
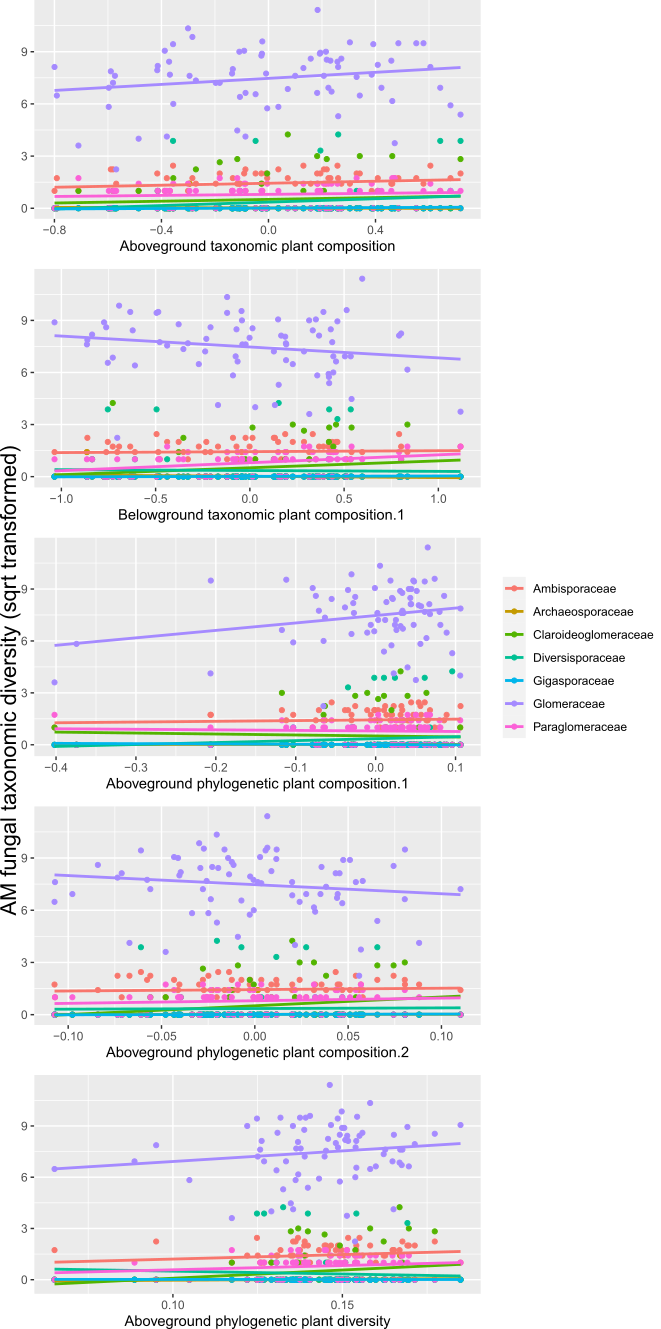
**

**Fig. S12** Effect of the spatial covariates on the arbuscular mycorrhizal fungal (AMF) taxonomic (a) and phylogenetic (b) diversity. We show the averaged parameter estimates (standardised regression coefficients) of model predictors and the associated 95% confident intervals. Abbreviations: dbMEM, distance-based Moran’s eigenvectors maps.


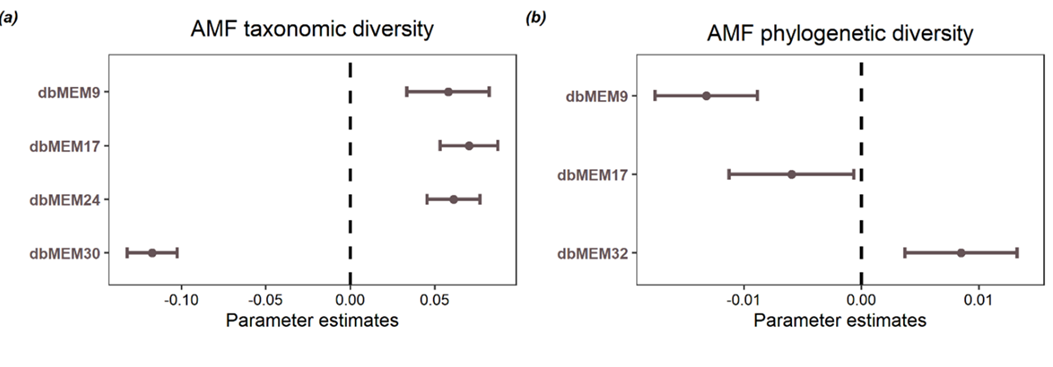


**Table. S1** List of plant species found in our study plot

| **Family** | **Genus** | **Species** | **Abbreviations** |
| --- | --- | --- | --- |
| Apiaceae | *Bupleurum* | *Bupleurum fruticescens* | Bup.fru |
|  | *Eryngium* | *Eryngium campestre* | Ery.cam |
| Asteraceae | *Helichrysum* | *Helichrysum serotinum* | Hel.ser |
|  | *Jurinea* | *Jurinea humilis* | Jur.hum |
|  | *Leuzea* | *Leuzea conifera* | Leu.con |
|  | *Santolina* | *Santolina chamaecyparissus* | San.cha |
|  | *Staehelina* | *Staehelina dubia* | Sta.dub |
| Boraginaceae | *Lithodora* | *Lithodora fruticosa* | Lit.fru |
| Brassicaceae | *Matthiola* | *Matthiola fruticulosa* | Mat.fru |
| Caprifoliaceae | *Cephalaria* | *Cephalaria leucantha* | Cep.leu |
| Caryophyllaceae | *Arenaria* | *Arenaria cavanillesiana* | Are.cav |
| Cistaceae | *Fumana* | *Fumana ericoides* | Fum.eri |
|  |  | *Fumana thymifolia* | Fum.thy |
|  | *Helianthemum* | *Helianthemum cinereum* | Hel.cin |
|  |  | *Helianthemum hirtum* | Hel.hir |
|  |  | *Helianthemum syriacum* | Hel.syr |
| Euphorbiaceae | *Euphorbia* | *Euphorbia nicaeensis* | Eup.nic |
| Fabaceae | *Coronilla* | *Coronilla minima* | Cor.min |
|  | *Hippocrepis* | *Hippocrepis commutata* | Hip.com |
|  | *Ononis* | *Ononis tridentata* | Ono.tri |
| Fagaceae | *Quercus* | *Quercus coccifera* | Que.coc |
|  |  | *Quercus rotundifolia* | Que.rot |
|  |  | *Quercus* sp. | Que.sp |
| Lamiaceae | *Lavandula* | *Lavandula latifolia* | Lav.lat |
|  | *Phlomis* | *Phlomis lychnitis* | Phl.lyc |
|  | *Salvia* | *Salvia lavandulifolia* | Sal.lav |
|  | *Sideritis* | *Sideritis hirsuta* | Sid.hir |
|  | *Sideritis* | *Sideritis incana* | Sid.inc |
|  | *Teucrium* | *Teucrium capitatum* | Teu.cap |
|  |  | *Teucrium gnaphalodes* | Teu.gna |
|  |  | *Teucrium* sp. | Teu.sp |
|  | *Thymus* | *Thymus* sp. | Thy.sp |
| Linaceae | *Linum* | *Linum narbonense* | Lin.nar |
|  |  | *Linum suffruticosum* | Lin.suf |
| Poaceae | *Avenula* | *Avenula bromoides* | Ave.bro |
|  | *Koeleria* | *Koeleria vallesiana* | Koe.val |
|  | *Stipa* | *Stipa pennata* | Sti.pen |
|  |  | *Stipa* sp*.* | Sti.sp |
|  |  | *Stipa tenacissima* | Sti.ten |
| Primulaceae | *Coris* | *Coris monspeliensis* | Cor.mon |
| Rosaceae | *Sanguisorba* | *Sanguisorba minor* | San.min |
| Rubiaceae | *Asperula* | *Asperula aristata* | Asp.ari |
| Santalaceae | *Thesium* | *Thesium divaricatum* | The.div |
| Thymelaeaceae | *Thymelaea* | *Thymelaea pubescens* | Thy.pub |

**Table S2** Summary statistics for soil physicochemical properties. Mean, standard deviation (SD), minimum (min) and maximum (max) values, and coefficient of variation (CV) of 11 soil physicochemical variables for the 83 soil samples. Abbreviations: Gluc, β-glucosidase (µmol/gr dry soil/h); Phos, acid phosphatase activity (µmol/gr dry soil/h); SOC, soil organic carbon (%); N, soil total nitrogen (mg/g soil); P, soil available phosphorus (mg/g soil); K, potassium content (mg/g soil); Cond, electric conductivity (µS/cm).

| Statistic | Gluc | Phos | SOC | N | P | K | pH | Cond | Sand | Silt | Clay |
| --- | --- | --- | --- | --- | --- | --- | --- | --- | --- | --- | --- |
|  | (µmol/gr/h) | (µmol/gr/h) | (%) | (mg/g) | (mg/g) | (mg/g) | - | (µS/cm) | (%) | (%) | (%) |
| Mean | 0.74 | 0.52 | 1.21 | 0.65 | 0.11 | 0.01 | 8.19 | 84.26 | 39.91 | 49.05 | 11.04 |
| SD | 0.41 | 0.24 | 0.30 | 0.27 | 0.04 | 0.00 | 0.12 | 29.80 | 3.36 | 2.22 | 1.96 |
| Min | 0.09 | 0.16 | 0.65 | 0.06 | 0.00 | 0.01 | 7.70 | 40.60 | 30.70 | 44.42 | 7.82 |
| Max | 2.26 | 1.40 | 2.29 | 1.51 | 0.21 | 0.02 | 8.41 | 198.30 | 46.40 | 55.01 | 17.49 |
| CV | 0.55 | 0.45 | 0.25 | 0.42 | 0.39 | 0.27 | 0.01 | 0.35 | 0.08 | 0.05 | 0.18 |

**Table S3** List of arbuscular mycorrhizal fungi virtual taxa (VT) in the MaarjAM database found in our study plot indicating taxonomic placement (order, family and genus), included known species or unidentified cultures.

| **Order** | **Family** | **Virtual taxon (VT)** | **Genus** | **Included known species and unidentified cultures** |
| --- | --- | --- | --- | --- |
|  |  |  |  |  |
| Archaeosporales | Ambisporaceae | VTX00242 | *Ambispora* | *Ambispora callosa* |
|  |  |  |  | *Ambispora leptoticha* |
|  |  |  |  | *Ambispora sp.* |
|  |  | VTX00283 |  | *Ambispora Amb* |
|  |  |  |  | *Ambispora gerdemannii* |
|  |  |  |  | *Ambispora Torrecillas12b Amb1* |
|  |  |  |  |  |
|  | Archaeosporaceae | VTX00245 | *Archaeospora* | *Archaeospora sp.* |
|  |  | VTX00338 |  | *Archaeospora Aca* |
|  |  |  |  |  |
| Diversisporales | Diversisporaceae | VTX00062 | *Diversispora* | *Diversispora Diversispora1* |
|  |  | VTX00353 |  | *Diversispora MO* |
|  |  |  |  |  |
|  | Gigasporaceae | VTX00049 | *Scutellospora* | *Scutellospora Liu2012b Phylo* |
| Glomerales | Glomeraceae | VTX00063 | *Glomus* | *Glomus NES04* |
|  |  |  |  | *Glomus sp.* |
|  |  |  |  | *Glomus Torrecillas12b Glo G15* |
|  |  | VTX00064 |  | *Glomus NF21* |
|  |  |  |  | *Glomus sp.* |
|  |  |  |  | *Glomus Torrecillas12b Glo G9* |
|  |  | VTX00067 |  | *Glomus Liu2012b Phylo* |
|  |  | VTX00072 |  | *Glomus Ligrone07* |
|  |  | VTX00092 |  | *Glomus Liu2012b Phylo* |
|  |  | VTX00093 |  | *Glomus sp.* |
|  |  | VTX00103 |  | *Glomus Alguacil09b Glo G6* |
|  |  |  |  | *Glomus sp.* |
|  |  | VTX00113 |  | *Glomus Glo G5* |
|  |  |  |  | *Glomus sp. 8451.1* |
|  |  | VTX00114 |  | *Glomus irregulare* |
|  |  | VTX00125 |  | *Glomus Liu2012b Phylo* |
|  |  |  |  | *Glomus Wirsel OTU10* |
|  |  | VTX00130 |  | *Glomus Alguacil09b Glo G7* |
|  |  |  |  | *Glomus sp.* |
|  |  | VTX00135 |  | *Glomus Glom 1B.8* |
|  |  | VTX00149 |  | *Glomus Alguacil12b GLO G11* |
|  |  |  |  | *Glomus Alguacil12b GLO G13* |
|  |  | VTX00151 |  | *Glomus NES14* |
|  |  |  |  | *Glomus NES31* |
|  |  | VTX00153 |  | *Glomus sp.* |
|  |  | VTX00160 |  | *Glomus sp.* |
|  |  | VTX00163 |  | *Glomus Ligrone07* |
|  |  |  |  | *Glomus sp.* |
|  |  | VTX00191 |  | *Glomus Alguacil12b GLO G14* |
|  |  |  |  | *Glomus sp.* |
|  |  | VTX00199 |  | *Glomus sp.* |
|  |  |  |  | *Glomus Torrecillas12b Glo G21* |
|  |  | VTX00199 |  | *Glomus Wirsel OTU7* |
|  |  | VTX00214 |  | *Glomus Liu2012b Phylo* |
|  |  |  |  | *Glomus NES18* |
|  |  |  |  | *Glomus sp.* |
|  |  | VTX00222 |  | *Glomus indicum* |
|  |  |  |  | *Glomus Liu2012b Phylo* |
|  |  |  |  | *Glomus sp.* |
|  |  | VTX00295 |  | *Glomus Glo* |
|  |  | VTX00342 |  | *Glomus sp.* |
|  |  |  |  | *Glomus Torrecillas12b Glo G18* |
|  |  |  |  | *Glomus Torrecillas12b Glo G20* |
|  |  | VTX00393 |  | *Glomus Liu2012b Phylo* |
|  |  | VTX00407 |  | *Glomus Alguacil12b GLO G12* |
|  |  | VTX00409 |  | *Glomus Torrecillas12b Glo G13* |
|  |  | VTX00418 |  | *Glomus Glo G4* |
|  |  |  |  |  |
|  | Claroideoglomeraceae | VTX00055 | *Claroideoglomus* | *Claroideoglomus sp.* |
|  |  | VTX00193 |  | *Claroideoglomus Glo* |
|  |  |  |  | *Claroideoglomus Glo G6* |
|  |  |  |  | *Claroideoglomus lamellosum* |
|  |  |  |  | *Claroideoglomus sp.* |
|  |  |  |  | *Claroideoglomus Torrecillas12b Glo G1* |
|  |  |  |  |  |
| Paraglomerales | Paraglomeracea | VTX00001 | *Paraglomus* | *Paraglomus sp.* |
|  |  | VTX00308 |  | *Paraglomus Glom 1B.13* |
|  |  |  |  | *Paraglomus Para2* |
|  |  | VTX00335 |  | *Paraglomus Alguacil12b PARA3* |
|  |  |  |  | *Paraglomus ma+E3:E76jewskii* |
|  |  |  |  |  |

**Table S4** ANOVA-like results based on redundancy analysis (RDA) testing the effect of the forward-selected spatial covariates on the arbuscular mycorrhizal fungal (AMF) taxonomic and phylogenetic composition. The F-ratio-like statistic was tested using the Monte Carlo test based on 999 permutations. ***, P < 0.001; **, P < 0.01; *, P < 0.05.

| Predictor set | | |  | AMF taxonomic composition | | | | | AMF phylogenetic composition | | | | |
| --- | --- | --- | --- | --- | --- | --- | --- | --- | --- | --- | --- | --- | --- |
|  | Predictor |  |  | Monte Carlo test | | | |  | Monte Carlo test | | | |  |
|  |  |  |  | F-ratio |  | *p* |  |  | F-ratio |  | *p* |  |  |
| Spatial covariates | | |  |  |  |  |  |  |  |  |  |  |  |
|  | x |  |  | 1.29 |  | 0.076 |  |  | 1.65 |  | 0.189 |  |  |
|  | y |  |  | 1.69 |  | 0.007 | ** |  | 1.81 |  | 0.142 |  |  |
|  | dbMEM13 |  |  | 1.48 |  | 0.017 | * |  | 6.72 |  | 0.002 | ** | |
|  | dbMEM14 |  |  | 1.71 |  | 0.002 | ** |  |  |  |  |  |  |
|  | dbMEM15 |  |  |  |  |  |  |  | 2.71 |  | 0.054 |  |  |
|  | dbMEM30 |  |  |  |  |  |  |  | 4.85 |  | 0.010 | ** | |

**Table S5** Results of the AICc-based model selection based on linear models testing the response of arbuscular mycorrhizal fungal (AMF) taxonomic and phylogenetic diversity to taxonomic (a-b) and phylogenetic (c-d) plant community attributes controlling for the effects of aboveground plant cover, root biomass, soil physicochemical properties and the distance-based Moran’s eigenvectors maps (dbMEM). Only models which differed from the best model in less than 2 AICc units are shown. Abbreviations: AT, aboveground taxonomic; BT, belowground taxonomic; AP, aboveground phylogenetic; BP, belowground phylogenetic; Composition.1 and composition.2, are variables calculated as the scores of each sample on the two first axes of nonmetric multidimensional scaling (nMDS; Fig. 1); SOC, soil organic carbon; AICc, Corrected Akaike Information Criterion; df, degree of freedom; ΔAIC, delta of the best selected models (AICc < 2).

| a) AMF taxonomic diversity |  |  |
| --- | --- | --- |
| Predictors |  | models |
| (Intercept) |  | 4.12 |
| AT composition.1 |  | 0.08 |
| BT composition.1 |  | -0.09 |
| Root biomass |  | 0.07 |
| dbMEM3 |  | -0.12 |
| dbMEM9 |  | 0.08 |
| dbMEM17 |  | 0.04 |
| Fertility |  | -0.06 |
| df |  | 8 |
| AICc |  | 854.00 |
| delta |  |  |

| b) AMF phylogenetic diversity | | |  |  |  |  |  |  |  |  |  |  |  |  |  |  |  |  |  |  |  |  |  |
| --- | --- | --- | --- | --- | --- | --- | --- | --- | --- | --- | --- | --- | --- | --- | --- | --- | --- | --- | --- | --- | --- | --- | --- |
| Predictors | models | |  |  |  |  |  |  |  |  |  |  |  |  |  |  |  |  |  |  |  |  |  |
| (Intercept) | -2.05 | -2.05 | -2.05 | -2.05 | -2.05 | -2.05 | -2.05 | -2.05 | -2.05 | -2.05 | -2.05 | -2.05 | -2.05 | -2.05 | -2.05 | -2.05 | -2.05 | -2.05 | -2.05 | -2.05 | -2.05 | -2.05 | -2.05 |
| AT diversity |  |  | -0.06 |  | -0.06 |  |  | -0.06 |  |  |  |  |  | -0.05 |  |  |  |  |  |  | -0.03 |  |  |
| Plant cover |  |  |  |  |  |  |  |  |  |  |  |  |  |  |  |  | -0.03 |  | -0.04 |  |  |  |  |
| BT composition.1 | 0.07 | 0.07 | 0.06 | 0.06 | 0.07 | 0.06 | 0.07 | 0.07 | 0.06 |  | 0.07 | 0.07 | 0.06 |  | 0.07 |  | 0.07 | 0.07 | 0.06 | 0.08 | 0.08 | 0.07 | 0.07 |
| AT composition.2 |  |  |  |  |  |  |  |  |  |  |  |  |  |  | -0.03 |  |  |  |  |  |  | -0.03 |  |
| dbMEM16 | 0.06 | 0.06 |  | 0.05 | 0.05 |  | 0.06 |  |  |  |  | 0.06 |  |  | 0.07 | 0.05 | 0.06 | 0.06 | 0.06 | 0.06 | 0.06 | 0.07 | 0.06 |
| dbMEM17 | -0.08 | -0.08 | -0.09 | -0.09 | -0.09 | -0.09 | -0.09 | -0.09 | -0.09 | -0.09 | -0.08 | -0.08 | -0.08 | -0.10 | -0.08 | -0.09 | -0.08 | -0.08 | -0.09 | -0.08 | -0.08 | -0.08 | -0.08 |
| dbMEM25 |  |  |  |  |  |  |  |  |  |  |  | 0.04 |  |  |  |  |  | 0.03 |  |  |  |  |  |
| dbMEM30 | 0.05 |  |  |  |  |  | 0.05 | 0.04 | 0.05 |  | 0.05 | 0.05 |  |  | 0.05 |  | 0.05 |  |  |  | 0.05 |  | 0.06 |
| dbMEM9 | -0.09 | -0.09 | -0.08 | -0.08 | -0.08 | -0.08 | -0.08 | -0.08 | -0.08 | -0.07 | -0.09 | -0.09 | -0.09 | -0.07 | -0.09 | -0.07 | -0.10 | -0.09 | -0.09 | -0.09 | -0.09 | -0.09 | -0.09 |
| Root biomass |  |  |  |  |  |  |  |  |  |  |  |  |  |  |  |  |  |  |  | -0.03 |  |  |  |
| Texture |  |  |  |  |  |  |  |  |  |  |  |  |  |  |  |  |  |  |  |  |  |  | 0.03 |
| Basic pH | -0.10 | -0.11 | -0.12 | -0.11 | -0.12 | -0.11 | -0.11 | -0.11 | -0.10 | -0.11 | -0.10 | -0.10 | -0.10 | -0.12 | -0.10 | -0.12 | -0.10 | -0.10 | -0.11 | -0.11 | -0.10 | -0.10 | -0.10 |
| BT diversity |  |  | 0.08 | 0.05 | 0.07 | 0.06 | 0.05 | 0.08 | 0.05 | 0.07 |  |  |  | 0.09 |  | 0.06 |  |  | 0.06 |  |  |  |  |
| df | 8 | 7 | 8 | 8 | 9 | 7 | 9 | 9 | 8 | 6 | 7 | 9 | 6 | 7 | 9 | 7 | 9 | 8 | 9 | 8 | 9 | 8 | 9 |
| AICc | 36.20 | 36.41 | 36.42 | 36.53 | 36.54 | 36.76 | 36.95 | 37.21 | 37.27 | 37.32 | 37.44 | 37.61 | 37.61 | 37.66 | 37.78 | 37.81 | 37.84 | 37.85 | 37.86 | 37.91 | 37.97 | 37.99 | 38.05 |
| delta | 0.00 | 0.21 | 0.22 | 0.34 | 0.34 | 0.56 | 0.75 | 1.01 | 1.08 | 1.13 | 1.25 | 1.41 | 1.42 | 1.46 | 1.58 | 1.61 | 1.64 | 1.65 | 1.66 | 1.71 | 1.78 | 1.80 | 1.86 |

| c) AMF taxonomic diversity | | |
| --- | --- | --- |
| Predictors |  | model |
| (Intercept) |  | 4.122 |
| AP diversity |  | 0.104 |
| Plant cover |  | 0.074 |
| AP composition.2 |  | -0.101 |
| dbMEM1 |  | 0.0825 |
| dbMEM9 |  | 0.0806 |
| Root biomass |  | 0.070 |
| Fertility |  | -0.061 |
| df |  | 8 |
| AICc |  | 928.91 |
| delta |  |  |

| d) AMF phylogenetic diversity | | | |  |  |  |
| --- | --- | --- | --- | --- | --- | --- |
| Predictors |  | models | |  |  |  |
| (Intercept) |  | -2.05 | -2.05 | -2.05 | -2.05 | -2.05 |
| AP diversity |  |  |  | -0.04 |  |  |
| AP composition.1 |  | -0.07 | -0.07 | -0.08 | -0.07 |  |
| AP composition.2 |  | 0.09 | 0.08 | 0.10 | 0.09 | 0.09 |
| dbMEM17 |  | -0.08 | -0.08 | -0.09 | -0.07 | -0.09 |
| dbMEM9 |  | -0.08 | -0.09 | -0.08 | -0.08 | -0.08 |
| Root biomass |  |  |  |  | -0.03 |  |
| Basic pH |  | -0.10 | -0.10 | -0.10 | -0.10 | -0.10 |
| BP diversity |  |  | 0.04 |  |  |  |
| df |  | 7 | 8 | 8 | 8 | 6 |
| AICc |  | 31.5 | 32.6 | 33.1 | 33.3 | 33.5 |
| delta |  | 0 | 1.06 | 1.53 | 1.79 | 1.92 |
